# Factors influencing work ability and return-to-work in individuals affected by post-COVID: a systematic review

**DOI:** 10.1186/s12889-026-27839-7

**Published:** 2026-05-21

**Authors:** Marcel Ottiger, Iris Poppele, Anchalee Sarah Seefen Soliman, Torsten Schlesinger, Katrin Müller

**Affiliations:** Department of Social Science of Physical Activity and Health, Institute of Human Movement Science and Health, Faculty of Behavioural and Social Sciences, Chemnitz University of Technology, Chemnitz, 09107 Germany

**Keywords:** COVID-19, Post-COVID, Post-acute sequelae of SARS-CoV-2 (PASC), Work ability, Return-to-work, Occupational status, Employment, Facilitators, Obstacles

## Abstract

**Background:**

Post-COVID is associated with prolonged impairments in work ability and return-to-work (RTW). The heterogeneity and complexity of post-COVID symptoms present major obstacles to a sustainable RTW. This systematic review aims to identify facilitators and obstacles affecting work ability and RTW.

**Methods:**

Eligible studies examined factors affecting work ability or RTW in post-COVID patients. Systematic search of literature was performed up to March 2025 using MEDLINE, CENTRAL, PsycINFO, Scopus, and Web of Science. Study selection followed the Preferred Reporting Items for Systematic Review and Meta-analysis Statement. Risk of bias was evaluated with the “Joanna Briggs Institute Critical Appraisal Tools”.

**Results:**

31 studies published between 2021 and 2025 were included in the analysis. Most originated from Europe and North America with sample sizes reaching from small qualitative studies to large registry-based cohort studies. The identified factors (*N* = 59; facilitators: *n* = 25, obstacles: *n* = 34) could be grouped into four domains: Disease-related factors associated with SARS-CoV-2 infection (*n* = 8), Individual biopsychosocial factors (*n* = 35), Contextual workplace factors (*n* = 10), Healthcare system and service-related factors (*n* = 6). The most frequently reported obstacles were fatigue and neurocognitive impairments, stigmatization, lack of managerial support, and rigid RTW policies. Adequate workplace adjustments, interprofessional therapeutic interventions, and self-management strategies facilitate work ability and RTW.

**Conclusions:**

Work ability and RTW with post-COVID is determined by complex multilevel interactions of biopsychosocial, workplace-related, and systemic factors. Findings suggest that coordinated care and workplace adaptations may help to bridge the gap between medical recovery and occupational participation. Future research should aim to better understand how multiple factors interact in individual cases to develop targeted, evidence-based interventions and policy frameworks.

**PROSPERO registration number:**

CRD420251010826.

**Supplementary Information:**

The online version contains supplementary material available at 10.1186/s12889-026-27839-7.

## Background

The COVID-19 pandemic has left behind not only acute health burdens but also a growing number of individuals struggling with long-term consequences that extend far beyond the initial SARS-CoV-2 infection. Among these, post-COVID poses a particularly complex and persistent challenge. For those affected, ongoing symptoms can severely limit daily functioning, social participation, and, critically, the ability to work. This creates urgent demands on healthcare system and employers to develop effective care structures to facilitate return-to-work (RTW) and restoration of long-term workforce resilience [1].

Post-COVID is defined as a clinical condition in which symptoms persist or newly emerge beyond 12 weeks after an acute SARS-CoV-2 infection [2, 3]. Common complaints include fatigue, reduced physical and cognitive performance, psychological symptoms, as well as sleep disturbances and pain [4–7]. Even individuals with initially mild COVID-19 can experience lasting impairments, with prevalence estimates around 6.5% among all infected individuals [8]. Recovery is often incomplete over months, and for many, prognosis remains uncertain [9].

### Impact of post-COVID on work ability and return-to-work

Beyond the medical burden, post-COVID frequently impairs everyday functioning and occupational participation. Basic activities such as household chores may become overwhelming, leisure pursuits are often reduced, and social interactions decline [10]. Professionally, the consequences are severe: many affected individuals are unable to work for extended periods, while others return with reduced capacity [11]. Studies have documented long-term work incapacity, reduced working hours, and the need for workplace adjustments even after RTW [12–18].

Understanding sustainable RTW with post-COVID requires consideration of two interrelated concepts: work ability and RTW. Work ability describes an individual’s capacity to meet job demands, resulting from the interaction between personal resources and workplace conditions [19]. RTW is a holistic, multifactorial systemic process that involves coordination between medical providers, rehabilitation services, employers, and the employees themselves, and is commonly operationalized as an outcome (e.g., return to employment or time to return) [20, 21]. Accordingly, in this review, RTW is understood as both a dynamic process and an outcome, indicating whether an individual has resumed employment. Personal influencing factors such as positive attitude and social support, as well as organizational conditions and workplace interventions, influence RTW after prolonged work incapacity, although evidence on their effectiveness in the context of post-COVID remains inconsistent [21, 22].

In the context of post-COVID, early findings suggest that, beyond organizational aspects, neurocognitive impairments and psychological symptoms play a critical role in reducing work ability [23, 24]. The fluctuating nature and individual course of post-COVID often coincide with considerable psychosocial distress, which constitutes a major obstacle to successful RTW [25]. Even after an initial RTW and apparent improvement, many individuals continue to experience recurring problems with energy levels and concentration, as well as post-exertional malaise, which severely impact their daily functioning and occupational performance [17, 18, 26–28]. Moreover, employees commonly report an increase in pressure from the work environment due to their inability to perform fully [26]. According to a qualitative study, all of the patients interviewed (*N* = 12) began to reflect on and reorient their professional identity during the recovery process. They gradually shifted from a self-concept that was strongly performance-driven to one that prioritized health and realistic expectations [29].

The uncertainty regarding return to the workplace, combined with persistent symptoms, can result in feelings of helplessness, stigmatization, and financial strain [30]. Beyond these individual challenges and obstacles, post-COVID also affects labor markets and economic productivity. Estimates suggested that post-COVID resulted in a reduction in labor supply in 2022 of up to 0.5%, primarily due to absenteeism and reduced work capacity [31]. Against this background, Nagra et al. [28] highlighted the need for multidimensional and context-sensitive approaches to promote sustainable RTW in post-COVID populations. These preliminary insights underscore the need for further research to capture the dynamic interplay of personal, occupational and systemic factors influencing work ability and RTW with post-COVID.

### Research gap and study objectives

Despite an increasing number of studies on post-COVID, significant knowledge gaps remain regarding its impact on occupational participation, as well as the adequacy of diagnosis, therapeutic options, and care structures [3]. The heterogeneity and fluctuating nature of post-COVID symptoms present particular obstacles for maintaining or regaining work ability [11, 32]. While early findings highlight considerable work limitations among affected individuals, systematic investigations into the RTW process and its influencing factors remain scarce. Existing research predominantly focused on qualitative assessments of workplace-related support measures (e.g., [33]), often without a systematic analysis of biopsychosocial, work-related, and sociodemographic influences and their interrelations. Our previous systematic review [1] primarily quantified overall RTW rates and work-related outcomes, while the underlying determinants and their multilevel interactions across domains received less attention. A comprehensive and structured synthesis of current research on facilitators and obstacles of work ability and RTW in post-COVID patients is therefore lacking.

The aim of this systematic review is to examine the current state of research on biopsychosocial, workplace-related, and sociodemographic factors influencing work ability and RTW of individuals with post-COVID. This knowledge base is intended to inform future approaches in clinical practice, workplace support (managing of vocational rehabilitation), and policy-making to better assist individuals affected by post-COVID in regaining and maintaining their occupational roles.

### Theoretical framework: the international classification of functioning, disability and health (ICF)

To conceptualize the multidimensional influences on work ability and RTW identified in the literature, this review is theoretically grounded in the International Classification of Functioning, Disability and Health (ICF) developed by the World Health Organization [34]. The ICF offers a biopsychosocial framework that conceptualizes health and disability as outcomes of the dynamic interaction between a person’s health condition, contextual factors, and the corresponding environment. Rather than focusing solely on medical diagnoses, the ICF integrates biological, psychological, and social dimensions, thereby enabling a holistic multilevel analysis of functioning and participation [35, 36]. According to the ICF, the individual health condition is determined by three core components: (1) body functions and structures, (2) activities, and (3) participation, which are influenced by contextual factors at environmental (e.g., workplace, healthcare system) and personal (e.g., age, education) level. This multidimensional perspective aligns with current evidence highlighting the complex interplay between disease-related, individual, occupational, and systemic determinants of RTW in post-COVID populations [37]. Applying the ICF model as a theoretical framework enables the structured and deductive categorization of factors influencing work ability and RTW.

## Methods

This systematic review was conducted in accordance with the Preferred Reporting Items for Systematic Reviews and Meta-Analyses (PRISMA) Statement (see Supplementary Table S1) [38]. The review protocol was registered in the International Prospective Register of Systematic Reviews (PROSPERO) database (Registration number: CRD420251010826).

### Search strategy

Following the PRISMA guidelines, a comprehensive search was performed in multiple electronic databases considered relevant to the topic. The following databases were searched from January 2020 to March 2025: MEDLINE (via PubMed), Cochrane Central Register of Controlled Trials (CENTRAL), PsycINFO, Scopus, and Web of Science. The search strategy combined Medical Subject Headings (MeSH) and free-text terms related to “post-COVID”, “return-to-work”, “work ability”, and associated factors influencing work ability and RTW (see full search strategy in Supplementary Table S2).

In addition to database searching, a manual search of the reference lists of included studies and relevant reviews was conducted. Citation tracking was also performed to identify further potentially eligible studies. Study registers were searched to identify ongoing or unpublished studies. The search was limited to peer-reviewed articles published in English or German. The literature search was conducted independently by two reviewers to ensure comprehensiveness.

### Eligibility criteria

Studies were considered eligible if they examined adult individuals (≥ 18 years) with confirmed, suspected, or self-reported SARS-CoV-2 infection who developed post-COVID, defined as the persistence of symptoms for more than 12 weeks following an acute SARS-CoV-2 infection [39]. Eligible studies had to investigate factors influencing work ability, work productivity or the process of RTW in the context of post-COVID. No restrictions were placed on gender, occupational background or the severity of acute infection and the type of exposure or intervention, as long as the study focused on identifying factors associated with work ability or RTW. Eligible study designs included observational (e.g., cohort, cross-sectional) and interventional studies (e.g., randomized controlled trials), as well as qualitative research.

Studies were excluded if they were systematic reviews, meta-analyses, clinical guidelines, case series, case reports, pilot studies, scoping reviews, editorials, or commentaries, or if they did not involve human participants. Further exclusions applied to studies that did not examine work-related outcomes in the context of post-COVID, did not meet the required symptom duration of at least 12 weeks, or were published in languages other than English or German.

### Study selection

All search results were first imported into the reference management software EndNote, where duplicate records were removed. The remaining references were then exported to Rayyan as review management platform [40].

**Fig. 1:**
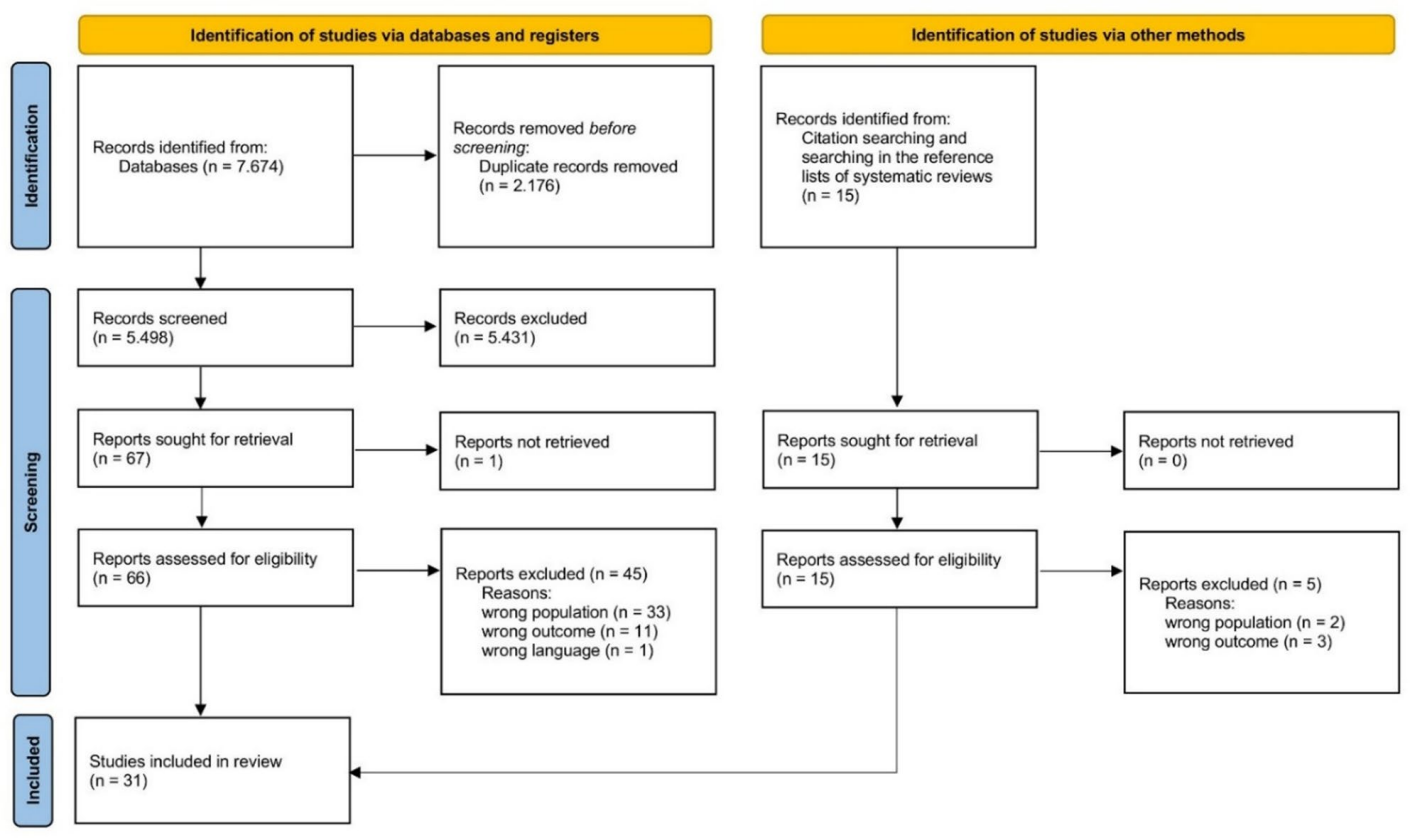
PRISMA flowchart of the article selection process

Two reviewers independently screened all records’ titles and abstracts to assess their potential eligibility by evaluating them against the predefined eligibility criteria. Next, two reviewers independently assessed the full texts of all potentially relevant studies. This included a detailed review of the study population, design, and reported outcomes to ensure that all inclusion criteria were met. The reasons for excluding studies were documented. Discrepancies in the screening or full-text assessment were resolved through discussion between the reviewers. If no agreement could be reached, a third reviewer was consulted to reach a final decision. The entire selection process was documented in a PRISMA flow diagram (see Fig. 1).

### Data extraction

Data extraction was conducted using a standardized Excel-based form that was initially piloted by the reviewers to ensure consistency. From each included study, the following information was extracted: authors, year of publication, country, study design, setting, sample size, population characteristics (e.g., age, gender, severity of the acute illness), type of intervention or healthcare service, presence and type of comparison group(s), and follow-up period. Furthermore, all reported influencing factors were extracted and categorized as disease-related, individual, workplace-related, or healthcare-related.

Two reviewers independently extracted the data. Both reviewers shared similar backgrounds and prior knowledge of post-COVID research and consistently applied the eligibility criteria throughout the extraction process. Any discrepancies were discussed and resolved through consensus, ensuring a high level of agreement throughout the extraction process.

### Risk of bias

The methodological quality and risk of bias of all included studies were assessed using the “Joanna Briggs Institute” (JBI) Critical Appraisal Tools, which provide standardized criteria tailored to different study designs [41]. Specifically, we applied the JBI Critical Appraisal Checklist for Cohort Studies [42], Cross-sectional Studies [42], and Qualitative Research [43].

Two reviewers independently performed the quality appraisal for each included study. Each item on the checklist was rated as “yes,” “no,” “unclear,” or “not applicable” based on the information reported, but no overall numeric score was assigned, in line with best practice for risk of bias assessments [41]. Any discrepancies between the reviewers were resolved through discussion; in cases where consensus could not be reached, a third reviewer was consulted. This procedure ensured a consistently high level of interrater agreement throughout the appraisal process. An overview of the appraisal tools is provided in Supplementary Table S3.

### Data synthesis

This review is conducted as a mixed-methods systematic review with a descriptive narrative synthesis. Given the considerable heterogeneity across the included studies in terms of design, population characteristics, outcome definitions of work ability and RTW, and the operationalization of independent variables, a meta-analysis was not feasible. Outcomes were assessed using different measures and time points, and effect estimates were reported in diverse formats. Even within potentially comparable subsets of studies, heterogeneity remained too high to allow for a meaningful quantitative synthesis. Instead, a narrative synthesis was conducted based on the Synthesis Without Meta-analysis guidelines [44].

The synthesis followed a structured multi-step approach. First, all reported factors related to work ability and RTW were extracted from the included studies. For quantitative studies, this included variables that were reported to be significantly associated with work ability or RTW, while for qualitative studies, factors were derived from reported themes and participant experiences. Second, the extracted factors were coded using a primarily deductive approach, with the ICF framework serving as the central analytical structure guiding the synthesis. This framework guided the development of the main categories, while allowing for minor inductive refinements where necessary. Third, similar factors were grouped into thematic categories reflecting conceptually related aspects. During this process, overlaps between categories were discussed within the research team and resolved through consensus. Finally, these categories were analytically synthesized into four overarching domains: (1) Disease-related factors associated with SARS-CoV-2 infection, (2) Individual biopsychosocial factors with the subdomains sociodemographic/ socio-economic factors, physical factors/ risk factors, psychological/ neuropsychological factors, social factors, and individual factors related to work ability and workplace, (3) Contextual workplace factors, and (4) Healthcare system and service-related factors. The four domains were conceptually aligned with the ICF framework. The assignment of factors to domains was based on their primary conceptual relevance, with explicit consideration of their functional role within the ICF model, acknowledging that some factors may span multiple domains. The synthesis reflects the frequency of reported factors across studies and does not imply equal strength of evidence. Findings from quantitative and qualitative studies were interpreted as complementary but representing different types of evidence and should not be interpreted as a proxy for methodological quality, which was assessed independently of study design.

## Results

### Search results and study selection

The systematic search of electronic databases yielded 7.674 records. After removal of duplicates and title-abstract screening, 67 full-text articles were assessed for eligibility. In addition, citation tracking and reference list screening identified further potentially relevant studies. Ultimately, 31 studies met the predefined inclusion criteria and were included in the final synthesis. The detailed study selection process is shown in the PRISMA flow diagram (see Fig. 1).

### Study characteristics

A total of 31 studies published between 2021 and 2025 were included in the final synthesis. Most of the studies were conducted in Europe and North America, with most originating from the UK (*n* = 8), Germany (*n* = 6), and the USA (*n* = 6).

The included studies employed various methodological approaches: *n* = 23 were observational (cohort studies: *n* = 11, cross-sectional studies: *n* = 12), and *n* = 8 used qualitative methods such as narrative or semi-structured interviews. The review did not include any interventional studies, as none were identified or met the predefined eligibility criteria.

Sample sizes ranged from 10 participants in small qualitative designs to 206.299 individuals in large registry-based cohort studies. The average sample size across all studies was approximately 8.411 participants. The populations studied primarily consisted of working-age adults with an overall mean age of 47 years and a predominance of female participants (Mean = 69%).

Study participants represented a wide range of occupational sectors, including healthcare, education, and public administration. Regarding infection verification, studies employed a mix of formal diagnostic criteria, self-reported diagnoses, and registry-confirmed infections. The time since acute infection varied considerably ranging from 3 to 30 months post-infection. The severity of the acute infection was often not explicitly reported; however, where stated, the majority of the cases were categorized as mild to moderate, with smaller proportions experiencing severe or critical illness.

The studies assessed work ability and RTW using a range of methods, including standardized instruments (e.g., Work Ability Index (WAI)), registry and survey data, as well as self-reported employment status and interview-based qualitative data.

An overview of the main characteristics of the included studies is provided in Table 1.

**Table 1:** Characteristics of the study population

Study	Country	Study design	Sample size (*N*)	Female Sex (%)	Mean (M) age (SD) or range	Follow-up time	Acute COVID-19 severity
Altmann et al. 2023 [45]	Germany	cohort study	42 (*n* = 21 PC)	12 (60.0)	N/A	admission and discharge	PC-group: 10 (48.0%) hospitalized 3 (14.0%) ICU
Anderson et al. 2025 [46]	UK	qualitative study	65	49 (75.3)	range: 20–65	N/A	N/A
Ayoubkhani et al. 2024 [47]	UK	cohort study	206.299 (*n* = 97.751 infected with SARS-CoV-2) (*n* = 8.440 PC)	group 1 (PC): 5.330 (63.2) group 2 (non-PC): 49.220 (55.1)	group 1 (PC): M: 46.3 (11.2) group 2 (non-PC): M: 44.3 (12.3)	monthly follow-up assessments (M: 12.3 assessments per participant)	N/A
Bonner & Ghouralal, 2024 [48]	USA	cross-sectional study	18.816 (17.8% PC-prevalence)	9.320 (49.5)	range: 18–65	N/A	N/A
Braig et al. 2024 [49]	Germany	cross-sectional study	9.572	5.548 (58.0)	M: 45.6 range: 18–65	N/A	7.236 (76.0%) no medical treatment 1.906 (20.0%) outpatient 273 (3.0%) inpatient 73 (1.0%) ICU
Brehon et al. 2022 [18]	Canada	cohort study	81	52 (64.0)	M: 48.9 (10.5)	pre and post rehabilitation	N/A
Chasco et al. 2022 [50]	USA	qualitative study	15	10 (66.7)	M: 49.3 range: 40–68	N/A	6 (40.0%) hospitalized 2 (13.3%) ICU
Delgado-Alonso et al. 2022 [12]	Spain	cross-sectional study	82	67 (87.0)	M: 46.3 (8.0)	N/A	15 (19.5%) hospitalized 3 (3.9%) ICU 4 (5.2%) ventilatory assistance
Diem et al. 2022 [15]	Switzerland	cross-sectional study	309	249 (80.6)	M: 44.6 range: 19–83	N/A	33 (10.7%) hospitalization 8 (2.6%) ICU
Ida et al. 2024 [51]	Brazil	cohort study	58	36 (62.0)	M: 52.8 (10.5)	12 months post symptom onset	39 (67%) hospitalized 35 (60.0%) ICU
Frisk et al. 2023 [52]	Norway	Cohort study	78	64 (82.0)	M: 40.3 (12.0)	12 months	64 (82.0%) mild/moderate 11 (14.0%) critical 3 (4.0%) severe
Green et al. 2023 [53]	UK	cross-sectional study	214	135 (63.0)	M: 51.0	N/A	26 (12.0%) hospitalized 7 (3.0%) ICU
Gyllensten et al. 2023 [24]	Sweden	qualitative study	19	13 (68.4)	M: 54.0 range: 29–63	N/A	N/A
Harvey-Dunstan et al. 2022 [54]	UK	cross-sectional study	42	28 (66.7)	M: 49.0 (10.0)	N/A	non-hospitalized COVID-19 sample
Jaber et al. 2025 [55]	Canada	cross-sectional study	2.726 (*n* = 1.031 PC)	group 1 (recovered): 1.068 (63.2) group 2 (PC): 791 (77.5)	group 1 (recovered): M: 45.1 (13.7) group 2 (PC): M: 45.9 (13.3)	N/A	78 (7.6%) hospitalized
Jebrini et al. 2025 [56]	Germany	Cohort study	259	group 1 (unable to work): 99 (60.7) group 2 (able to work): 63 (65.6)	group 1 (unable to work): M: 41.0 group 2 (able to work): M: 42.0	12 months	N/A
Kerksieck et al. 2023 [57]	Switzerland	cohort study	672	364 (54.2)	M: 42.1 (12.2)	12 months	10 (1.5%) hospitalized 662 (98.5%) non-hospitalized 1 (0.1%) ICU
Kisiel et al. 2023 [58]	Sweden	cross-sectional study	584 (*n* = 85 PC)	396 (67.8)	group 1 (non-hospitalized COVID): M: 44.2 (13.8) group 2 (hospitalized COVID): M: 58.5 (10.1) group 3 (PC): M: 50.0 (11.9)	N/A	119 (20.4%) hospitalized
LeGoff et al. 2023 [59]	USA	cross-sectional study	64	35 (54.7)	M: 48 (3.0)	N/A	N/A
Lunt et al. 2024 [60]	UK	qualitative study	10	10 (100)	*n* = 1 20–29 *n* = 1 30–39 *n* = 3 40–49 *n* = 1 49–50 *n* = 3 50–59 *n* = 1 ≥ 60 range: 25–63	N/A	N/A
MacEwan et al. 2025 [61]	USA	qualitative study	21	16 (76.0)	M: 47.6 range: 19–68	N/A	N/A
Miller et al. 2024 [62]	UK	qualitative study	25	17 (68.0)	M: 43.6 (14.7)	N/A	N/A
Müller et al. 2024 [37]	Germany	cohort study	114	86 (75.0)	M: 50.5 (10.9)	12 months	82 (72.0%) mild/moderate 27 (24.0%) severe 5 (4.0%) critical
Nielsen & Yarker 2024 [29]	UK	qualitative study	12	11 (92.0)	M: 45.0	N/A	N/A
Rutsch et al. 2023 [17]	Germany	cohort study	173	116 (68.2)	M: 52.5 (9.0)	12 months	82 (47.4%) mild 66 (38.2%) moderate 21 (12.1%) severe 4 (2.3%) critical
Saade et al. 2024 [63]	France	cross-sectional study	1.062	851 (80.0)	M: 41.0 range: 32–49	N/A	N/A
Stelson et al. 2023 [64]	USA	cross-sectional study	510	296 (58.0)	*n* = 32 18–29 *n* = 96 30-39 *n* = 115 40-49 *n* = 94 50–59 *n* = 58 ≥ 60 *n* = 115 no response	N/A	N/A
Strassburger et al. 2023 [65]	Germany	cross-sectional study	184	142 (77.0)	*n* = 13 18–29 or above 64 *n* = 105 30–49 *n* = 66 50–64	N/A	N/A
Venkatesh et al. 2024 [66]	USA	cohort study	2.928	1.878 (64.1)	M: 40.0 (12.6)	3 months	110 (3.8%) hospitalized
Walker et al. 2023 [67]	UK	cross-sectional study	3.754	2.675 (71.0)	M: 47.7 (12.3)	N/A	N/A
Westerlind et al. 2021 [68]	Sweden	cohort study	11.955	7.129 (59.6)	M: 48.0 (11.3)	4 months	N/A

*N* total sample size, *M *mean, *SD *standard deviation, *n *sample size, *PC *post-COVID, *N/A *not available, *ICU *Intensive Care Unit

### Risk of bias

The risk of bias was assessed separately for the included qualitative, cohort, and cross-sectional studies using established methodological appraisal tools. Overall, methodological quality across the included studies was acceptable to good, though it varied by study type.

Qualitative studies showed strong congruence between research questions, methodology, and data analysis. Semi-structured interviews and focus groups were effectively used to explore subjective experiences and reflections of individuals with post-COVID, particularly in relation to work and RTW processes. A major strength across studies was the rich and systematic representation of participant voices through illustrative quotations. Limitations included limited reflexivity regarding the researchers’ influence on the research process and interrater reliability was seldom reported.

Cohort studies demonstrated generally sound methodological design, with exposures (post-COVID vs. non-post-COVID) clearly defined, and follow-up periods were adequate. Strengths included use of standardized outcome measures and appropriate statistical analyses. Common limitations were inconsistent handling of confounders, variable reporting of baseline comparability, and insufficient strategies to address attrition or missing data. Non-comparative cohorts lacked an unexposed group, introducing potential bias.

Cross-sectional studies were typically well suited to descriptive and associative analyses with clearly defined inclusion criteria and valid outcome measurements, such as work ability or RTW status. Limitations included inconsistent control of confounding factors and reliance on self-reported exposure or outcomes, which may have introduced bias. Reporting quality varied, with some studies providing limited details on statistical adjustments or rationale for analytic approaches.

A comprehensive overview of the methodological appraisal for all included studies is presented in Supplementary Table S4.

### Influencing factors on work ability and return-to-work 

**Fig. 2:**
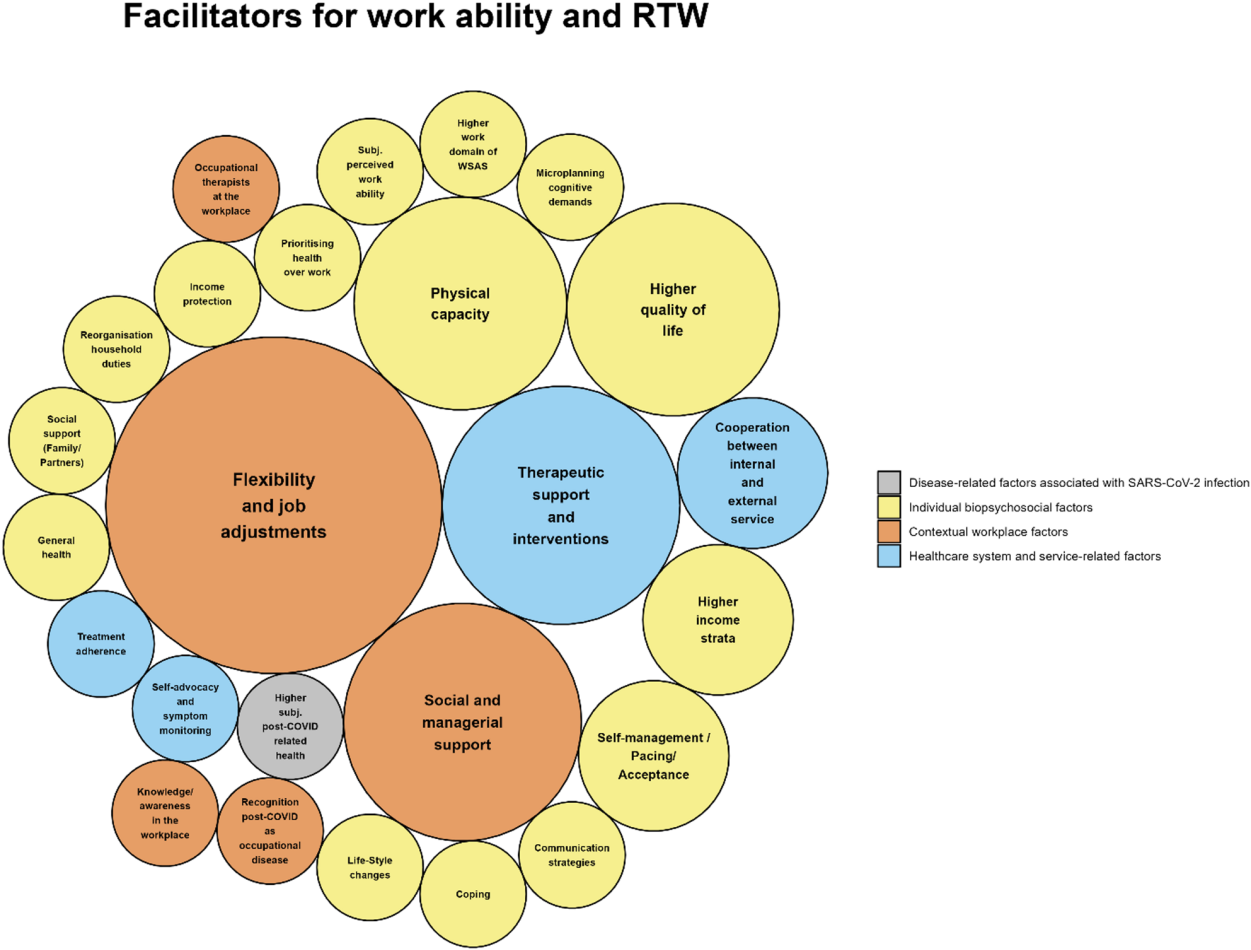
Facilitators for work ability and return-to-work visualized as bubble plot. The figure was created in R (version 4.4.2) using the packages ggplot2 and packcircles. Bubble area is proportional to number of studies reporting each factor, and colors indicate factor domains. See Table S5 in Supplement 1 for a list-wise description of facilitating factors

**Fig. 3:**
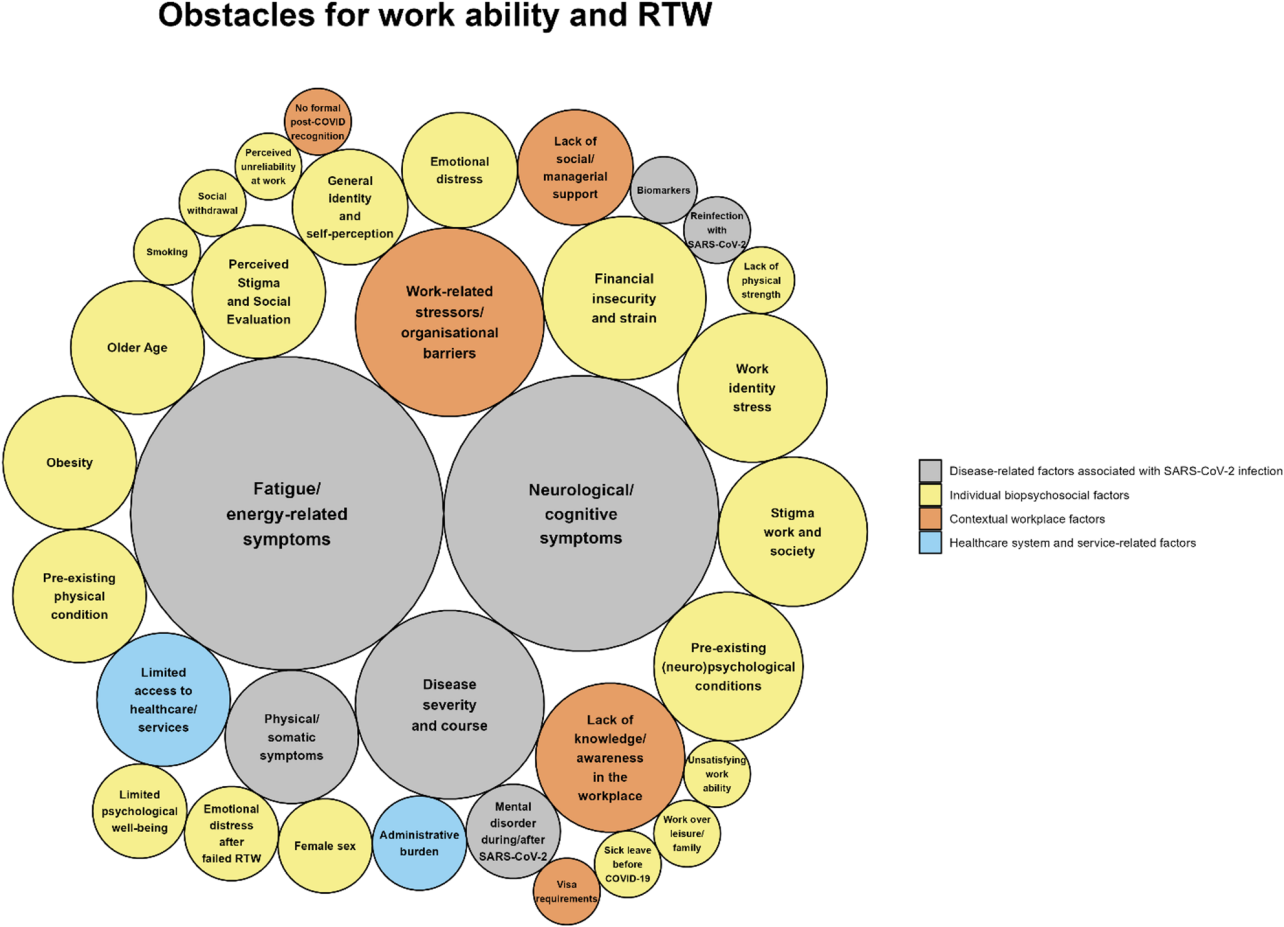
Obstacles for work ability and return-to-work visualized as bubble plot. The figure was created in R (version 4.4.2) using the packages ggplot2 and packcircles. Bubble area is proportional to number of studies reporting each factor, and colors indicate factor domains. See Table S5 in Supplement 1 for a list-wise description of obstacle factors

The synthesis of 31 included studies revealed *N* = 59 influencing factors affecting work ability and RTW among individuals with post-COVID. Of these, 25 were facilitators and 34 were obstacles. The factors were categorized into the four domains introduced in the Data synthesis section, which are conceptually aligned with key components of the ICF framework. Within the domain “Disease-related factors associated with SARS-CoV-2 infection”, eight factors were identified, one of which was classified as a facilitator and seven as obstacles. The domain “Individual biopsychosocial factors” with the subdomains sociodemographic/ socio-economic factors, physical factors/ risk factors, psychological/ neuropsychological factors, social factors, and individual factors related to work ability and workplace contains 35 influencing factors, 15 of which facilitating work ability and RTW while 20 act as obstacles. Within the domain “Contextual workplace factors”, ten factors were categorized, with five being facilitators and five being obstacles. The domain “Healthcare system and service-related factors” contains six influencing factors, of which four are facilitators and two are obstacles. A graphical representation of the facilitators and obstacles is provided in Figs. 2 and 3. The complete list of all identified factors is presented in Supplementary Table S5 for a well-structured overview of influencing factors.

#### Disease-related factors associated with SARS-CoV-2 infection

##### Facilitators

A higher subjective perceived physical and mental health related to post-COVID was positively associated with work ability and RTW [37].

##### Obstacles

The severity and course of disease are influencing factors on work ability and RTW. In particular, a longer time since infection was associated with lower RTW rates in some studies [47, 55]. Indicators of more severe disease, such as Intensive Care Unit (ICU) admission [49], inpatient treatment [49, 68] or hospitalization [55], were also identified as barriers. In addition, ongoing symptoms 12 months post infection with SARS-CoV-2 [51, 57], higher post-COVID symptom scores [58], and a higher number of concurrent symptoms [66] were consistently linked to reduced work ability and RTW. Further, persistent neurological and cognitive impairments, such as neurocognitive deficits [37, 45, 49], cognitive symptoms [12, 17, 29, 46, 56, 57, 60, 63, 67], brain fog [50, 53, 61, 62], and headaches or dizziness [49, 61, 62, 66], were common barriers. Among all reported symptoms, fatigue was the most frequently reported and disabling symptom [12, 15, 17, 24, 29, 37, 49, 50, 53, 54, 56, 61–63, 66, 67], often fluctuating [29, 46, 60, 64] and exacerbated by overexertion [46]. On the other hand, findings by Frisk et al. [52] indicated that work-related outcomes were not influenced by fatigue severity. Additional physical and somatic symptoms included chest discomfort [49], musculoskeletal pain [49, 66], anosmia/dysgeusia [49], breathlessness [62, 66], heart palpitations [62], and appetite disturbance [56]. Mental health symptoms, including depressive symptoms and anxiety [49, 67] also impeded work ability and RTW. Finally, biomarker abnormalities, such as elevated CRP, IL-6, leukocytes, neutrophils, triglycerides, lower HDL cholesterol and coagulation parameter changes, were linked to reduced work ability in one study [56]. Reinfection with SARS-CoV-2 was also reported as an additional obstacle for work ability and RTW [60].

### Individual biopsychosocial factors

#### Individual sociodemographic/ socio-economic factors

##### Facilitators

 Economic resources emerged as important facilitators for work ability and RTW, with income protection [60] and higher socioeconomic status [66, 68] being particularly relevant.

##### Obstacles

 Sociodemographic obstacles included female sex [49, 60], perimenopausal status [60] and older age [49, 56, 57, 68]. Furthermore, financial insecurity and concerns about an uncertain future, including employability, workability, and economic stability, hinder RTW among post-COVID patients. This includes financial concerns [46, 57, 60, 61, 64], income loss [50, 61], high medical costs, and lack of insurance coverage [50]. Statutory sick pay limits [46] and reduced insurance coverage [61] further exacerbated the financial concerns.

#### Individual physical factors/ risk factors

##### Facilitators

Better physical performance [37, 51, 65] and higher ratings of overall functional status [56] were positively associated with work ability and RTW. Further, better general health [55] is another facilitator for work ability and RTW.

##### Obstacles

 Conversely, reduced muscle strength [61] and pre-existing physical conditions [48, 49, 55, 60] were physical obstacles. In addition, risk factors such as current or former smoking [49] and obesity [48, 49, 56, 60] also compromised patients’ work ability and RTW.

#### Individual psychological factors/ neuropsychological factors

##### Facilitators

Several psychological resources and strategies were identified as facilitators of work ability and RTW. These included microplanning of cognitive demands [60], acquiring self-management strategies, including pacing and acceptance of functional limitations [29, 60], and health-related behavioural adaptations, such as lifestyle changes to mitigate physical risk factors [60]. In addition, effective communication strategies [60] and adaptive coping approaches [65] were identified as facilitators, too. In general, a higher quality of life is also positively associated with work ability and RTW of post-COVID patients [51, 53, 56, 65].

##### Obstacles

Psychological obstacles include pre-existing (neuro)psychological conditions, such as depression, anxiety disorders, and other mental health conditions [48, 49, 55, 57, 60]. A limited psychological well-being such as new or worsened psychiatric diagnoses [57] and higher depressive symptom levels [56] further diminishes work ability and RTW. Perceived stigma and social evaluation often led to symptom concealment [46, 50] and feelings of being scrutinized by others [50]. Performance pressure [61], and fear of negative judgement from others were frequently reported challenges [60]. Furthermore, disruptions in identity and self-perception emerged as significant barriers. Patients described a “spoiled identity” marked by shame, guilt, and self-doubt [46, 50, 62] and they find themselves in an identity conflict feeling neither sick nor well [46]. Affected patients also reported distress related to changes in social and occupational roles, particularly when previously independent individuals became dependent on support from others, which hindered successful RTW [46]. Other barriers included loss of daily routine [61] and feelings of failure and grief [46, 62].

#### Individual social factors

##### Facilitators

Social support from family or partners [64] and reorganization of household responsibilities [64] were enablers for work ability and RTW.

##### Obstacles

Social withdrawal [46] was identified as an obstacle for work ability and RTW in the context of social factors.

#### Individual factors related to work ability and workplace

##### Facilitators

A higher perceived work ability [17], higher scores on the Work and Social Adjustment Scale in the work domain [67] and prioritizing health over work [29] are the main facilitators within the individual factors related to work ability and workplace.

##### Obstacles

Perceived work-related stigmatization experiences [46, 50, 60, 61, 64] were a recurring obstacle. Further, work identity stress, a loss of their capable worker identity and of career aspirations [29, 46, 60–62] are significant barriers regarding work ability and RTW. One study highlights employees’ perception of being unreliable [62]. Additional individual, work-related obstacles are the emotional distress following redundancy or unsuccessful RTW attempts [46, 61], energy trade-offs prioritizing work over personal life [46], and dissatisfaction with one’s own work ability [56]. An association between pre-COVID sick leave and worse work ability was found in one study [68].

#### Contextual workplace factors

##### Facilitators

Contextual workplace factors played a central role in facilitating work ability and RTW. At a structural level, recognition and acceptance of post-COVID as an occupational illness [62] was identified as an important facilitator. Workplace-related key facilitators included supportive and trusting managers or employers [24, 46, 50], coworker support and an encouraging workplace culture that makes employees feel valued [24, 29, 46, 60], employer willingness to make accommodations [46], job security [60], and instrumental support [29].

Flexible work arrangements emerged as a key facilitating mechanism. These included adjusted tasks [24, 65], modified duties at admission [18], and task simplification [24]. Flexible hours and schedules [24, 46, 50, 60, 65], part-time work [29, 62, 64], and individually tailored phased RTW programs [60] were also beneficial.

Practical strategies such as cheat sheets or list-making tools [50, 61], frequent breaks [24, 50, 61, 62, 64], and remote or hybrid work models [24, 29, 50, 60, 62, 64] supported RTW. Quiet workspaces [62], phased or long-term planned RTW arrangements [24, 62], more hours spread across more days [60], and becoming self-employed related to autonomy and flexibility [60] further enhanced the process.

Additional facilitators included training for employers [62], which reflected greater work functioning and adaptability. In one study, the affected individuals expressed the necessity of access to occupational therapists at the workplace [62].

##### Obstacles

Structural barriers included visa restrictions that complicated employment prospects [46] and the absence of formal post-COVID recognition, which in some countries limited access to disability benefits [50].

Workplace-related barriers included a lack of management support [29, 46], managerial distrust [62], and feelings of isolation from workplace communities [29]. Insufficient workplace knowledge and understanding of COVID-19 was also reported [24, 60, 62, 64], which could lead to the withdrawal of previously granted accommodations, such as remote work, thereby hindering employees’ RTW [61].

Task-related stressors and organizational obstacles at work were common. These comprised rigid RTW policies [29, 46], and unrealistic performance expectations [46]. High multitasking demands [50] and a fast-paced work environment [50] placed additional strain on employees. Other reported barriers included post-work exhaustion [24], night shifts [57], and pressure to RTW prematurely [29, 61]. Logistical challenges such as long commutes [62] also hindered RTW. In addition, receiving generic rather than individualized RTW advice, particularly due to limited knowledge of post-COVID among professionals, was identified as a barrier [29]. Finally, specific work surroundings and tasks acted as barriers, including working with patients or pupils [29], screen-intensive work [29, 64], and job requirements involving dense reading or extensive writing [64].

#### Healthcare system and service-related factors

##### Facilitators

Access to occupational health services [46], neurocognitive screening and mental health care [59], occupational therapy [61], speech therapy [61], cognitive therapy [61], and workplace-based occupational therapists [62] supported RTW. Use of interprofessional long-COVID clinics [60], supportive healthcare providers, who enable sick leave and workers’ compensation by diagnosing post-COVID [64], and coordinated care between internal and external services [29, 60] were additional facilitators. Self-advocacy and symptom monitoring [46] and adherence to treatment plans [60] also played a crucial role.

##### Obstacles

Barriers related to the healthcare system included long delays between symptom onset and admission to an occupational rehabilitation program [18], a perceived need for additional support [53], difficult access to therapies [60, 61], and high administrative burden [46, 50].

## Discussion

The synthesis of 31 studies in this review provides a holistic perspective on the multifactorial determinants of work ability and RTW among individuals with post-COVID. When structured according to ICF, the identified factors span disease-related impairments, individual characteristics, workplace environment and structures, and healthcare system and service-related conditions. This multidimensional framework underscores that RTW is not a purely medical or organizational event, but the result of interacting biopsychosocial processes shaped by health, individual resources, behaviour and contextual conditions, consistent with guidelines recommending a biopsychosocial, and individualized RTW approach [69, 70]. In this sense, the findings reflect the biopsychosocial model embedded in the ICF and underline the importance of considering these interdependencies when addressing work participation. Within this framework, disease-related factors primarily represent impairments in body functions, while individual factors capture personal resources and coping processes. Workplace and healthcare system factors correspond to environmental influences that can either facilitate or hinder participation. Together, these components illustrate how limitations at the impairment level can translate into restrictions in work participation, depending on the surrounding context. This conceptual understanding is supported by recent empirical evidence. A mixed-methods study by Su et al. [71] demonstrates that sustainable employment among individuals with post-COVID is shaped by complex, multilevel factors. The study further highlights non-linear RTW trajectories, fluctuating and often invisible symptoms, and mismatches between functional capacity and job demands, as well as challenges related to workplace accommodations, stigma, and structural barriers. A notable gap concerns the activities component of the ICF. Most studies examine body functions (e.g., fatigue) and participation (e.g., employment status), while daily routines or physical activity are seldom addressed. However, qualitative findings indicate substantial limitations in daily activities e.g., household tasks or pacing energy across the day [28, 72]. Given that these activity-level limitations may directly affect the ability to sustain work demands and manage energy, integrating activity-based assessments into RTW research could provide promising additional insight into functional capacity between impairment and participation levels. This may contribute to develop more comprehensive and individualized recovery strategies aligned with the ICF Core Set for Vocational Rehabilitation [73].

Fatigue, post-exertional symptom exacerbation, and neurocognitive dysfunction represent major barriers to RTW, often persisting for months or years after infection. These factors clearly influence work-related executive functions [74, 75]. In line with this, recent quantitative evidence by Stigmar et al. [76] highlights mental fatigue as the strongest predictor of poor work ability, alongside physical fatigue and functional limitations in daily activities. Notably, only 18% of participants were on sick leave, suggesting a discrepancy between perceived work ability and formal work absence. While severe acute illness predicts poorer outcomes [77, 78], the time since infection does not consistently correlate with improved RTW, reflecting fluctuating and individualized recovery patterns [5], although possible selection biases in workplace-based studies should also be considered. Interestingly, some studies found that a longer time since infection was associated with lower RTW rates [47, 55]. Although this may seem counterintuitive, it probably indicates that individuals with a longer disease duration since infection tend to have more persistent or severe symptoms. In particular, prolonged disease duration may indicate a process of symptom chronification, characterized by ongoing impairments in physical and mental health, lower rates of physical activity and self-rated overall health [79]. A population-based cohort study by Ballouz et al. [80] demonstrated that individuals with post-COVID show persistently reduced work ability up to three years after infection, with only limited improvement over time. These findings reinforce the notion of prolonged and potentially chronic impairment trajectories affecting sustainable RTW.

Older age, female sex, and pre-existing conditions were associated with poorer RTW outcomes, aligning with evidence on risk factors for post-COVID and associated physical and cognitive impairments [81, 82]. Beyond the findings of the included studies, previous research has proposed potential explanations for the observed differences, particularly within specific subgroups such as women. These include hormonal influences, inflammatory processes, and social factors such as unequal care responsibilities [83]. However, these mechanisms were not directly examined in the included studies and should therefore be seen as hypotheses, which could be addressed in further studies.

Environmental and structural workplace factors played a pivotal role in either facilitating or hindering RTW. Supportive leadership, transparent communication, flexible schedules, task modifications, hybrid work models and individualized RTW arrangements were consistently described as effective facilitators. In contrast, rigid return policies, unrealistic performance expectations, and a low managerial understanding were key barriers. These findings align with occupational health recommendations [84] and guidance from the European Agency for Safety and Health at Work [85]. They demonstrate that environmental adaptations can mitigate functional limitations even when health impairments persist.

Psychological and social dimensions also play a key role. Stigma, both at the workplace and in society, was frequently reported and is supported by evidence. Stigmatization can exacerbate psychological symptoms [86], discourage help seeking [87–89], and even lead to patients abandoning medical care [30]. This aligns with broader research linking health-related stigma to reduced function and employment loss [90]. Beyond stigma, identity-related challenges were prominent. Many participants described disrupted occupational identity, with feelings of loss or inadequacy due to reduced work capacity and inability to meet previous role expectations [46, 61]. Such disruptions are linked to reduced participation and prolonged sickness absence [91]. Self-management strategies such as pacing and acceptance of performance limitations were frequently described as enabling factors when embedded within supportive work environments [29, 65]. Research shows that energy conservation, boundary setting and communication strategies can reduce functional problems and support RTW [92], although their success depends on organizational support [29]. Healthcare system and service-related factors were equally important. Access to interprofessional long-COVID clinics and therapies such as occupational or cognitive training promoted recovery [60, 61], whereas fragmented care structures [18, 53] long waiting times [60, 61] and administrative burdens [46, 50] were reported as barriers. Similarly, a qualitative study by Schmachtenberg et al. [93], not included in this review due to differing inclusion criteria, reported similar challenges in healthcare access and coordination. Within the ICF framework, these features represent environmental factors that can facilitate or constrain participation. Multimodal and digital rehabilitation concepts have been discussed in the literature as potentially offering flexible and scalable approaches to address complex post-COVID needs; however, their direct impact on RTW remains unclear and was not specifically examined in the included studies [37, 94]. Future intervention development should consider both biopsychosocial and contextual factors to promote sustainable work participation.

The findings of the systematic review underline the need for comprehensive labor and social protection policies to ensure financial security and promote the sustainable RTW of individuals affected by post-COVID at a societal level.

### Strengths and limitations

Several strengths and limitations should be considered. Strengths include long follow-up intervals reaching up to 30 months after SARS-CoV-2 infection. Furthermore, some of the included studies had large sample sizes, with over 200.000 participants. The systematic review included studies with both qualitative and quantitative measures, enabling conclusions to be drawn at both the individual and group levels. Lastly, the overall risk of bias assessment is generally acceptable. The limitations of the current systematic review include the heterogeneous assessment tools for workability and RTW used in the included studies. Some tools are validated (e.g., WAI), but self-reports and qualitative interviews were also used. The substantial heterogeneity across studies also highlights a need for greater standardization in future research. In particular, the limited number of studies using comparable outcome definitions, measurement instruments, and reporting formats currently restricts the feasibility of quantitative synthesis and meta-analysis. It is not always possible to generalize the results due to differences in working conditions, tasks and content (e.g., office jobs vs. working with patients), even if there is a broad range of professions within the selected studies. In addition, the identified factors reflect different types of evidence depending on the underlying study design. While quantitative studies provide evidence on statistical associations, qualitative studies offer context-specific insights into lived experiences. These differences should be considered when interpreting the relative importance of individual factors. No formal assessment of the certainty of evidence (e.g., using GRADE) was performed. Given the heterogeneity of study designs, outcomes, and measures, such an evaluation was not feasible, but this remains a limitation of the review. In addition, non-significant findings were not consistently reported across the included studies, as many studies focused on reporting selected or statistically significant results. This limited the possibility of systematically synthesizing factors not associated with work ability or RTW. Furthermore, heterogeneity between hospitalized and non-hospitalized populations could not be systematically examined, as many studies included mixed samples or did not clearly report hospitalization status. As these groups may differ substantially in terms of disease severity, symptom burden, and recovery trajectories, this limits the interpretation of work ability and RTW outcomes. Additionally, the included studies were predominantly conducted in Western countries, which may limit the generalizability of the findings to other regions, including countries in the Global South and Global East, as well as low- and middle-income settings. As the literature search was limited to studies published up to March 2025, more recent publications may not have been captured, which should be considered when interpreting the findings in the context of the rapidly evolving post-COVID literature. The assessment tools for the risk of bias do not provide a total score or rating, which would enable comparison with other studies. Furthermore, the risk of bias assessment is a subjective rating that can vary across depending on the rater. Lastly, the review is restricted to articles written in English or German.

### Implications for practice and policy

This review highlights the need for a biopsychosocial, work-oriented approach to rehabilitation and RTW of post-COVID patients, as reflected by the identified interaction of disease-related, individual, workplace, and healthcare system factors. Rather than targeting single factors in isolation, the findings suggest that effective RTW interventions should address three interrelated levels: (1) health-related impairments (e.g., fatigue, neurocognitive limitations) (2), individual coping capacities and self-management strategies, and (3) environmental and organizational conditions, particularly within the workplace. In line with the ICF framework, interventions may be most effective when combining these components, for example through coordinated approaches that integrate symptom management, support for self-regulation and pacing, and adaptations of job demands and work environments. This may help to reduce mismatches between functional capacity and work requirements and support sustainable work participation. Based on the reported barriers and facilitators, early, work-focused screening may help to assess cognitive load tolerance, orthostatic stability, and fatigue thresholds to define realistic workload limits. Sustainable RTW appears to require individualized, reversible plans with flexible adjustments of working hours and tasks, scheduled breaks, and clear stop criteria, as indicated by findings on symptom variability and work capacity limitations. Workplace-related facilitators identified in the review, such as task modification, reduced multitasking, quiet environments, and hybrid work models, suggest that adapting work demands is essential for supporting RTW. However, these examples are primarily derived from specific occupational contexts and may not be universally applicable to all types of work. Therefore, workplace adaptations should be tailored to individual needs, job characteristics, and broader work environments.

Furthermore, the importance of supportive leadership, communication, and reduced stigmatization reflects the role of social and organizational factors identified across studies. Similarly, findings related to fragmented care and access barriers underline the need for improved coordination between primary care, occupational health, and specialized services (e.g., occupational, cognitive, and speech therapy), as well as the importance of strengthening self-management and communication skills to support autonomy and sustainable work participation.

Finally, the identified gap in activity-level assessments suggests that integrating activity-based approaches may help to better capture functional limitations relevant for daily life and work participation. These implications are consistent with ICF principles, emphasizing that RTW depends on interactions between individual, environmental, and organizational factors.

Policy implications include clarifying legal frameworks for recognizing post-COVID as disability or occupational disease, securing benefits, and workplace protections. Further, financial security should be provided by structuring sick pay and transitional benefits in a way that does not penalize flexible or partial RTW. Employer-based initiatives, such as workplace rehabilitation, case management, and job coaching, should be supported. Standardizing work-relevant outcome measures (e.g., work hours, functional indices such as the WAI) would improve monitoring, comparability, and evidence-based policy development.

## Conclusion

This systematic review shows that work ability and RTW among individuals with post-COVID are determined by complex interactions of biopsychosocial, workplace-related, and systemic factors. Persistent fatigue and neurocognitive impairments represent key barriers, whereas flexible work arrangements, supportive leadership, and interprofessional rehabilitation facilitate sustainable RTW. Psychological and social dimensions, including stigma, disrupted occupational identity, and coping strategies, further influence RTW and highlight the need for comprehensive, context-sensitive support. Taken together, the identified barriers related to fragmented care, limited access to services, and workplace constraints suggest that improved coordination of interprofessional care pathways and workplace adaptations may help to bridge the gap between medical recovery and the demands of occupational participation. Future research should prioritize individualized analyses of RTW trajectories to inform the development of tailored, evidence-based interventions and policy frameworks.

## Supplementary Information

Supplementary Material 1.

## Data Availability

All data generated or analysed during this study are included in this published article and its supplementary information files.

## Abbreviations

ICF: International Classification of Functioning, Disability and Health
ICU: Intensive Care Unit
JBI: Joanna Briggs Institute
PC: post-COVID
PRISMA: Preferred Reporting Items for Systematic Reviews and Meta-Analyses
PROSPERO: International Prospective Register for Systematic Reviews
RTW: return-to-work
M: Mean
MeSH: Medical Subject Headings
N: total sample size
n: sample size
N/A: not available
SD: standard deviation
WAI: Work Ability Index
