# Supplementary material for: Factors influencing work ability and return-to-work in individuals affected by post-COVID: a systematic review
Source: BMC Public Health. 2026 May 21;26:1703. doi: 10.1186/s12889-026-27839-7 (PMC13200315; doi:10.1186/s12889-026-27839-7)
Supplement: Supplementary file 1 — Supplementary Material 1. [file 12889_2026_27839_MOESM1_ESM.docx]

**Supplementary material**

**Supplementary Table S1.** PRISMA checklist.

| **Section and Topic** | **Item #** | **Checklist item** | **Location where item is reported** |
| --- | --- | --- | --- |
| **TITLE** | | |  |
| Title | 1 | Identify the report as a systematic review. | p. 1 |
| **ABSTRACT** | | |  |
| Abstract | 2 | See the PRISMA 2020 for Abstracts checklist. | p. 2 |
| **INTRODUCTION** | | |  |
| Rationale | 3 | Describe the rationale for the review in the context of existing knowledge. | p. 3-4 |
| Objectives | 4 | Provide an explicit statement of the objective(s) or question(s) the review addresses. | p. 5 |
| **METHODS** | | |  |
| Eligibility criteria | 5 | Specify the inclusion and exclusion criteria for the review and how studies were grouped for the syntheses. | p. 7 |
| Information sources | 6 | Specify all databases, registers, websites, organisations, reference lists and other sources searched or consulted to identify studies. Specify the date when each source was last searched or consulted. | p. 6-7 |
| Search strategy | 7 | Present the full search strategies for all databases, registers and websites, including any filters and limits used. | Supplementary Table S2 |
| Selection process | 8 | Specify the methods used to decide whether a study met the inclusion criteria of the review, including how many reviewers screened each record and each report retrieved, whether they worked independently, and if applicable, details of automation tools used in the process. | p. 7-8 |
| Data collection process | 9 | Specify the methods used to collect data from reports, including how many reviewers collected data from each report, whether they worked independently, any processes for obtaining or confirming data from study investigators, and if applicable, details of automation tools used in the process. | p. 8-9 |
| Data items | 10a | List and define all outcomes for which data were sought. Specify whether all results that were compatible with each outcome domain in each study were sought (e.g. for all measures, time points, analyses), and if not, the methods used to decide which results to collect. | p. 8-9 |
|  | 10b | List and define all other variables for which data were sought (e.g. participant and intervention characteristics, funding sources). Describe any assumptions made about any missing or unclear information. | p. 8-9 |
| Study risk of bias assessment | 11 | Specify the methods used to assess risk of bias in the included studies, including details of the tool(s) used, how many reviewers assessed each study and whether they worked independently, and if applicable, details of automation tools used in the process. | p. 9 |
| Effect measures | 12 | Specify for each outcome the effect measure(s) (e.g. risk ratio, mean difference) used in the synthesis or presentation of results. | N/A |
| Synthesis methods | 13a | Describe the processes used to decide which studies were eligible for each synthesis (e.g. tabulating the study intervention characteristics and comparing against the planned groups for each synthesis (item #5)). | p. 9-11 |
|  | 13b | Describe any methods required to prepare the data for presentation or synthesis, such as handling of missing summary statistics, or data conversions. | p. 9-11 |
|  | 13c | Describe any methods used to tabulate or visually display results of individual studies and syntheses. | p. 9-11 |
|  | 13d | Describe any methods used to synthesize results and provide a rationale for the choice(s). If meta-analysis was performed, describe the model(s), method(s) to identify the presence and extent of statistical heterogeneity, and software package(s) used. | p. 9-11 |
|  | 13e | Describe any methods used to explore possible causes of heterogeneity among study results (e.g. subgroup analysis, meta-regression). | N/A |
|  | 13f | Describe any sensitivity analyses conducted to assess robustness of the synthesized results. | N/A |
| Reporting bias assessment | 14 | Describe any methods used to assess risk of bias due to missing results in a synthesis (arising from reporting biases). | N/A |
| Certainty assessment | 15 | Describe any methods used to assess certainty (or confidence) in the body of evidence for an outcome. | N/A |
| **RESULTS** | | |  |
| Study selection | 16a | Describe the results of the search and selection process, from the number of records identified in the search to the number of studies included in the review, ideally using a flow diagram. | Figure 1 |
|  | 16b | Cite studies that might appear to meet the inclusion criteria, but which were excluded, and explain why they were excluded. | Figure 1 |
| Study characteristics | 17 | Cite each included study and present its characteristics. | Table 1 |
| Risk of bias in studies | 18 | Present assessments of risk of bias for each included study. | Supplementary Table S4 |
| Results of individual studies | 19 | For all outcomes, present, for each study: (a) summary statistics for each group (where appropriate) and (b) an effect estimate and its precision (e.g. confidence/credible interval), ideally using structured tables or plots. | N/A |
| Results of syntheses | 20a | For each synthesis, briefly summarise the characteristics and risk of bias among contributing studies. | p. 11-21 |
|  | 20b | Present results of all statistical syntheses conducted. If meta-analysis was done, present for each the summary estimate and its precision (e.g. confidence/credible interval) and measures of statistical heterogeneity. If comparing groups, describe the direction of the effect. | N/A |
|  | 20c | Present results of all investigations of possible causes of heterogeneity among study results. | N/A |
|  | 20d | Present results of all sensitivity analyses conducted to assess the robustness of the synthesized results. | N/A |
| Reporting biases | 21 | Present assessments of risk of bias due to missing results (arising from reporting biases) for each synthesis assessed. | N/A |
| Certainty of evidence | 22 | Present assessments of certainty (or confidence) in the body of evidence for each outcome assessed. | N/A |
| **DISCUSSION** | | |  |
| Discussion | 23a | Provide a general interpretation of the results in the context of other evidence. | p. 21-24 |
|  | 23b | Discuss any limitations of the evidence included in the review. | p. 24-26 |
|  | 23c | Discuss any limitations of the review processes used. | p. 24-26 |
|  | 23d | Discuss implications of the results for practice, policy, and future research. | p. 26-27 |
| **OTHER INFORMATION** | | |  |
| Registration and protocol | 24a | Provide registration information for the review, including register name and registration number, or state that the review was not registered. | p. 6 |
|  | 24b | Indicate where the review protocol can be accessed, or state that a protocol was not prepared. | p. 6 |
|  | 24c | Describe and explain any amendments to information provided at registration or in the protocol. | N/A |
| Support | 25 | Describe sources of financial or non-financial support for the review, and the role of the funders or sponsors in the review. | p. 29 |
| Competing interests | 26 | Declare any competing interests of review authors. | p. 29 |
| Availability of data, code and other materials | 27 | Report which of the following are publicly available and where they can be found: template data collection forms; data extracted from included studies; data used for all analyses; analytic code; any other materials used in the review. | p. 29 |

**Supplementary Table S2.** Search strategy of the literature.

**PubMed**

**Date of search: 27.03.2025**

**# 1** (coronavirus[MeSH Terms]) OR (covid-19[MeSH Terms]) OR (coronavirus infections[MeSH Terms]) OR (Severe acute respiratory syndrome-related coronavirus[MeSH Terms]) OR (Post-Acute COVID-19 Syndrome[MeSH Terms])

**# 2** (covid[Title/Abstract]) OR (covid-19[Title/Abstract]) OR (coronavirus*[Title/Abstract]) OR (corona virus*[Title/Abstract]) OR (2019-ncov[Title/Abstract]) OR (sars-cov-2[Title/Abstract]) OR (cov-19[Title/Abstract]) OR (covid 19[Title/Abstract]) OR (covid19[Title/Abstract]) OR (severe acute respiratory syndrome coronavirus 2[Title/Abstract]) OR (coronavirus infection*[Title/Abstract]) OR (covid infection*[Title/Abstract]) OR (post COVID-19[Title/Abstract]) OR (post-acute COVID-19[Title/Abstract]) OR (post-acute COVID-19 syndrome[Title/Abstract]) OR (Long COVID[Title/Abstract]) OR (Post-COVID-19[Title/Abstract]) OR (Chronic COVID-19[Title/Abstract]) OR (Long-haul COVID-19[Title/Abstract]) OR (post-acute sequelae of SARS-CoV-2 infection[Title/Abstract]) OR (post-acute sequelae of COVID-19[Title/Abstract])

**# 3** (workplace[MeSH Terms]) OR (Occupational health[MeSH Terms]) OR (work performance[MeSH Terms]) OR (work capacity evaluation[MeSH Terms]) OR (sick leave[MeSH Terms]) OR (employment[MeSH Terms])

**# 4** (ability to work[Title/Abstract]) OR (work abilit*[Title/Abstract]) OR (capacity to work[Title/Abstract]) OR (work* capacity[Title/Abstract]) OR (work outcome*[Title/Abstract]) OR (work participation[Title/Abstract]) OR (labor participation[Title/Abstract]) OR (employabil*[Title/Abstract]) OR (productivity[Title/Abstract]) OR (workplace[Title/Abstract]) OR (work place*[Title/Abstract]) OR (return to work[Title/Abstract]) OR (return-to-work[Title/Abstract]) OR (back to work[Title/Abstract]) OR (sickness absence[Title/Abstract]) OR (sick leave[Title/Abstract]) OR (disability leave[Title/Abstract]) OR (occupational health[Title/Abstract]) OR (reintegration[Title/Abstract]) OR (re-integration[Title/Abstract]) OR (work reintegration[Title/Abstract])

**# 5** (Risk Factors[MeSH Terms]) OR (Protective Factors[MeSH Terms]) OR (Precipitating Factors[MeSH Terms])

**# 6** (factor*[Title/Abstract]) OR (determinant*[Title/Abstract]) OR (barrier*[Title/Abstract]) OR (facilitator*[Title/Abstract]) OR (predictor*[Title/Abstract]) OR (enabler*[Title/Abstract]) OR (Modifier*[Title/Abstract]) OR (contributing factor*[Title/Abstract]) OR (inhibitor*[Title/Abstract])

**# 7** #1 OR #2

**# 8** #3 OR #4

**# 9** #5 OR #6

**# 10** #7 AND #8 AND #9

**#11** (2020/1/1:2025/3/27[pdat])

**#12** (english[Filter]) OR (german[Filter])

**#13** #11 AND #12

**#14** #10 AND #13 NOT "Review"[Publication Type]

**Results = 2705 records**

**CENTRAL**

**Date of search: 27.03.2025**

**#1** MeSH descriptor: [COVID-19] explode all trees

**#2** MeSH descriptor: [Coronavirus] explode all trees

**#3** MeSH descriptor: [SARS-CoV-2] explode all trees

**#4** MeSH descriptor: [Coronavirus Infections] explode all trees

**#5** MeSH descriptor: [Severe acute respiratory syndrome-related coronavirus] explode all trees

**#6** MeSH descriptor: [Post-Acute COVID-19 Syndrome] explode all trees

**#7** #1 OR #2 OR #3 OR #4 OR #5 OR #6

**#8** (covid):ti,ab,kw OR (covid-19):ti,ab,kw OR (coronavirus*):ti,ab,kw OR (corona virus*):ti,ab,kw OR (2019 ncov):ti,ab,kw OR (sars-cov-2):ti,ab,kw OR (cov-19):ti,ab,kw OR (covid 19):ti,ab,kw OR (covid19):ti,ab,kw OR (severe acute respiratory syndrome coronavirus 2):ti,ab,kw OR (coronavirus infection*):ti,ab,kw OR (covid infection*):ti,ab,kw OR (post COVID-19):ti,ab,kw OR (post-acute COVID-19):ti,ab,kw OR (post-acute COVID-19 syndrome):ti,ab,kw OR (Long COVID):ti,ab,kw OR (Post-COVID-19):ti,ab,kw OR (Chronic COVID-19):ti,ab,kw OR (Long-haul COVID-19):ti,ab,kw OR (post-acute sequelae of SARS-CoV-2 infection):ti,ab,kw OR (post-acute sequelae of COVID-19):ti,ab,kw

**#9** #7 OR #8

**#10** MeSH descriptor: [Workplace] explode all trees

**#11** MeSH descriptor: [Occupational Health] explode all trees

**#12** MeSH descriptor: [Work Performance] explode all trees

**#13** MeSH descriptor: [Work Capacity Evaluation] explode all trees

**#14** MeSH descriptor: [Sick Leave] explode all trees

**#15** MeSH descriptor: [Employment] explode all trees

**#16** MeSH descriptor: [Return to Work] explode all trees

**#17** #10 OR #11 OR #12 OR #13 OR #14 OR #15 OR #16

**#18** (workplace*):ti,ab,kw OR (work place*):ti,ab,kw OR (occupational health):ti,ab,kw OR (work performance):ti,ab,kw OR (work capacity evaluation):ti,ab,kw OR (sick leave):ti,ab,kw OR (employment):ti,ab,kw OR (return to work):ti,ab,kw OR (ability to work):ti,ab,kw OR (work abilit*):ti,ab,kw OR (capacity to work):ti,ab,kw OR (work* capacity):ti,ab,kw OR (work outcome*):ti,ab,kw OR (work participation):ti,ab,kw OR (labor participation):ti,ab,kw OR (employabil*):ti,ab,kw OR (productivity):ti,ab,kw OR (return-to-work):ti,ab,kw OR (back to work):ti,ab,kw OR (sickness absence):ti,ab,kw OR (sick leave):ti,ab,kw OR (disability leave):ti,ab,kw OR (occupational health):ti,ab,kw OR (reintegration):ti,ab,kw OR (re-integration):ti,ab,kw OR (work reintegration):ti,ab,kw

**#19** #17 OR #18

**#20** MeSH descriptor: [Risk Factors] explode all trees

**#21** MeSH descriptor: [Protective Factors] explode all trees

**#22** #20 OR #21

**#23** (risk factor*):ti,ab,kw OR (protective factor*):ti,ab,kw OR (Precipitating Factor*):ti,ab,kw OR (factor*):ti,ab,kw OR (determinant*):ti,ab,kw OR (barrier*):ti,ab,kw OR (facilitator*):ti,ab,kw OR (predictor*):ti,ab,kw OR (enabler*):ti,ab,kw OR (modifier*):ti,ab,kw OR (contributing factor*):ti,ab,kw OR (ihibitor*):ti,ab,kw

**#24** #22 OR #23

**#25** #9 AND #19 AND #24 with Publication Year from 2020 to 2025, in Trials

**Results = 411 records**

**APA PsycInfo**

**Date of search: 27.03.2025**

**#1** **MA** "Coronavirus" OR **MA** "COVID-19" OR **MA** "Severe Acute Respiratory Syndrome" OR **MA** "Post-COVID-19 Conditions"

OR (TI coronavirus* OR AB coronavirus*) OR (TI covid-19 OR AB covid-19) OR (TI "Severe Acute Respiratory Syndrome" OR AB "Severe Acute Respiratory Syndrome") OR (TI "Post-COVID-19 Conditions" OR AB "Post-COVID-19 Conditions" ) OR (TI covid OR AB covid) OR (TI corona virus* OR AB corona virus*) OR (TI 2019-ncov OR AB 2019-ncov) OR (TI sars-cov-2 OR AB sars-cov-2) OR (TI cov-19 OR AB cov-19) OR (TI covid 19 OR AB covid 19) OR (TI covid19 OR AB covid19) OR (TI severe acute respiratory syndrome coronavirus 2 OR AB severe acute respiratory syndrome coronavirus 2) OR (TI coronavirus infection* OR AB coronavirus infection*) OR (TI covid infection* OR AB covid infection*) OR (TI post COVID-19 OR AB post COVID-19) OR (TI post-acute COVID-19 OR AB post-acute COVID-19) OR (TI post-acute COVID-19 syndrom OR AB post-acute COVID-19 syndrom) OR (TI Long COVID OR AB Long COVID) OR (TI Post-COVID-19 OR AB Post-COVID-19) OR (TI Chronic COVID-19 OR AB Chronic COVID-19) OR (TI Long-haul COVID-19 OR AB Long-haul COVID-19) OR (TI post-acute sequelae of SARS-CoV-2 infection OR AB post-acute sequelae of SARS-CoV-2 infection) OR (TI post-acute sequelae of COVID-19 OR AB post-acute sequelae of COVID-19)

**AND**

**#2 MA** “Organizational and Workplace Assessment” OR **MA** “Workplace Intervention” OR **MA** “Workplace Health and Well Being Measures” OR **MA** “Workplace environment” OR **MA** “Employee Well Being“ OR **MA** “Occupational health” OR **MA** “Job Performance” OR **MA** employment OR **MA** “Return to Work” OR **MA** Reemployment OR **MA** “Occupational Adjustment” OR **MA** productivity OR MA reintegration

OR (TI “Organizational and Workplace Assessment” OR AB “Organizational and Workplace Assessment”) OR (TI “Workplace Intervention” OR AB “Workplace Intervention”) OR (TI “Workplace Health and Well Being Measures” OR AB “Workplace Health and Well Being Measures”) OR (TI “Workplace environment” OR AB “Workplace environment”) OR (TI “Employee Well Being“ OR AB “Employee Well Being“) OR (TI “Occupational health” OR AB “Occupational health”) OR (TI “Job Performance” OR AB “Job Performance”) OR (TI employment OR AB employment) OR (TI “Return to Work” OR AB “Return to Work”) OR (TI Reemployment OR AB reemployment) OR (TI “Occupational Adjustment” OR AB “Occupational Adjustment”) OR (TI productivity OR AB productivity) OR (TI reintegration OR AB reintegration) OR (TI workplace OR AB workplace) OR (TI work place OR AB work place) OR (TI work performance OR AB work performance) OR (TI work capacity evaluation OR AB work capacity evaluation) OR (TI “sick leave” OR AB “sick leave”) OR (TI return-to-work OR AB return-to-work) OR (TI ability to work OR AB ability to work) OR (TI work abilit* OR AB work abilit*) OR (TI capacity to work OR AB capacity to work) OR (TI work* capacity OR AB work* capacity) OR (TI work outcome* OR AB work outcome*) OR (TI work participation OR AB work participation) OR (TI labor participation OR AB labor participation) OR (TI employabil* OR AB employabil*) OR (TI “back to work” OR AB “back to work”) OR (TI “sickness absence” OR AB “sickness absence”) OR (TI “disability leave” OR AB “disability leave”) OR (TI re-integration OR AB re-integration) OR (TI work reintegration OR AB work reintegration)

**AND**

**#3** **MA** Risk factors OR **MA** Protective Factors

OR (TI Risk factors OR AB Risk factors) OR (TI Protective Factors OR AB Protective Factors) OR (TI Precipitating Factors OR AB Precipitating Factors) OR (TI factor* OR AB factor*) OR (TI determinant*OR AB determinant*) OR (TI barrier* OR AB barrier*) OR (TI facilitator* OR AB facilitator*) OR (TI predictor* OR AB predictor*) OR (TI enabler* OR AB enabler*) OR (TI Modifier* OR AB Modifier*) OR (TI contributing factor* OR AB contributing factor*) OR (TI inhibitor* OR AB inhibitor*)

**#4** Filter: english, german, 01.01.2020-27.03.2025

**Results = 1.189 records**

**Scopus**

**Date of search: 27.03.2025**

**#1** (INDEXTERMS (Severe acute respiratory syndrome coronavirus 2) OR INDEXTERMS (long COVID) OR INDEXTERMS (post covid-19 functional status)) OR (TITLE-ABS-KEY(“Severe acute respiratory syndrome coronavirus 2” OR "long COVID” OR “post covid-19 functional status” OR “Severe acute respiratory syndrome coronavirus 2” OR “post-acute COVID-19" OR "post COVID-19" OR "chronic COVID-19" OR "Long-haul COVID-19" OR “post-acute sequelae of COVID-19" OR “post covid-19 condition” OR PCC))

AND

**#2** (INDEXTERMS (workplace) OR INDEXTERMS (“return to work”) OR INDEXTERMS (“occupational health”) OR INDEXTERMS (“job performance”) OR INDEXTERMS (“work capacity”) OR INDEXTERMS (employment) OR INDEXTERMS (medical leave) OR INDEXTERMS (work reintegration) OR TITLE-ABS-KEY(workplace OR "occupational health" OR "work performance" OR "work capacity" OR "sick leave" OR employment OR "return to work" OR "return-to-work" OR "ability to work" OR "work ability" OR "capacity to work" OR "work capacity" OR "work outcome" OR "work participation" OR "labor participation" OR employability OR "productivity" OR "back to work" OR "sickness absence" OR "disability leave" OR "reintegration" OR "work reintegration"))

AND

**#3** (INDEXTERMS (“risk factor”) OR INDEXTERMS (protection)) OR (TITLE-ABS-KEY("risk factor" OR "protective factor" OR "determinant" OR "barrier" OR "facilitator" OR "predictor" OR "enabler" OR "modifier" OR "contributing factor" OR "inhibitor"))

AND

**#4** PUBYEAR > 2019 AND PUBYEAR < 2026 AND ( LIMIT-TO ( DOCTYPE,"ar" ) OR LIMIT-TO ( DOCTYPE,"ch" ) OR LIMIT-TO ( DOCTYPE,"cp" ) OR LIMIT-TO ( DOCTYPE,"bk" ) OR LIMIT-TO ( DOCTYPE,"sh" ) OR LIMIT-TO ( DOCTYPE,"tb" ) )

**Results = 855 records**

**Web of Science**

**Date of search: 27.03.2025**

**#1** TS=(coronavirus OR covid-19 OR "coronavirus infection*" OR "Severe acute respiratory syndrome-related coronavirus" OR "Post-Acute COVID-19 Syndrome" OR sars-cov-2 OR "post COVID-19" OR "post-acute COVID-19" OR "post-acute COVID-19 syndrome" OR "Long COVID" OR "Post-COVID-19" OR "Chronic COVID-19" OR "Long-haul COVID-19" OR "post-acute sequelae of SARS-CoV-2 infection" OR "post-acute sequelae of COVID-19")

AND

**#2** TS=(workplace OR "Occupational health" OR "work performance" OR "work capacity evaluation" OR "sick leave" OR employment OR "ability to work" OR "work abilit*" OR "capacity to work" OR "work* capacity" OR "work outcome*" OR "work participation" OR "labor participation" OR employabil* OR productivity OR "return to work" OR "return-to-work" OR "back to work" OR "sickness absence" OR "sick leave" OR "disability leave" OR "occupational health" OR reintegration OR "work reintegration")

AND

**#3** TS=("Risk Factors" OR "Protective Factors" OR "Precipitating Factors" OR barrier* OR facilitator* OR predictor* OR contributing factor*)

Refined By: Languages: English or German. Publication Years: 2025 or 2024 or 2023 or 2022 or 2021 or 2020

**Results = 2514 records**

**Supplementary Table S3.** JBI Critical Appraisal Tools used for Risk of Bias (Lockwood et al., 2015; Moola et al., 2020).

| **JBI Critical Appraisal Checklist for Qualitative Research** | |
| --- | --- |
| ***Number*** | ***Question*** |
| Q1 | Is there congruity between the stated philosophical perspective and the research methodology? |
| Q2 | Is there congruity between the research methodology and the research question or objectives? |
| Q3 | Is there congruity between the research methodology and the methods used to collect data? |
| Q4 | Is there congruity between the research methodology and the representation and analysis of data? |
| Q5 | Is there congruity between the research methodology and the interpretation of results? |
| Q6 | Is there a statement locating the researcher culturally or theoretically? |
| Q7 | Is the influence of the researcher on the research, and vice- versa, addressed? |
| Q8 | Are participants, and their voices, adequately represented? |
| Q9 | Is the research ethical according to current criteria or, for recent studies, and is there evidence of ethical approval by an appropriate body? |
| Q10 | Do the conclusions drawn in the research report flow from the analysis, or interpretation, of the data? |
| **JBI Critical Appraisal Checklist for Cohort Studies** | |
| ***Number*** | ***Question*** |
| Q1 | Were the two groups similar and recruited from the same population? |
| Q2 | Were the exposures measured similarly to assign people to both exposed and unexposed groups? |
| Q3 | Was the exposure (post-COVID) measured in a valid and reliable way? |
| Q4 | Were confounding factors identified? |
| Q5 | Were strategies to deal with confounding factors stated? |
| Q6 | Were the groups/participants free of the outcome at the start of the study (or at the moment of exposure)? |
| Q7 | Were the outcomes (work ability/RTW) measured in a valid and reliable way? |
| Q8 | Was the follow up time reported and sufficient to be long enough for outcomes to occur? |
| Q9 | Was follow up complete, and if not, were the reasons to loss to follow up described and explored? |
| Q10 | Were strategies to address incomplete follow up utilized? |
| Q11 | Was appropriate statistical analysis used? |
| **JBI Critical Appraisal Checklist for Cross-sectional studies** | |
| ***Number*** | ***Question*** |
| Q1 | Were the criteria for inclusion in the sample clearly defined? |
| Q2 | Were the study subjects and the setting described in detail? |
| Q3 | Was the exposure (post-COVID) measured in a valid and reliable way? |
| Q4 | Were objective, standard criteria used for measurement of the condition? |
| Q5 | Were confounding factors identified? |
| Q6 | Were strategies to deal with confounding factors stated? |
| Q7 | Were the outcomes (work ability/RTW) measured in a valid and reliable way? |
| Q8 | Was appropriate statistical analysis used? |

**Supplementary Table S4.** Overview of methodological quality and risk of bias according to JBI Critical Appraisal Tools.

| **JBI Critical Appraisal Checklist for Qualitative Research** | | | | | | | | | | | |
| --- | --- | --- | --- | --- | --- | --- | --- | --- | --- | --- | --- |
| ***Authors*** | ***Q1*** | ***Q2*** | ***Q3*** | ***Q4*** | ***Q5*** | ***Q6*** | ***Q7*** | ***Q8*** | ***Q9*** | ***Q10*** |  |
| Anderson  et al. 2025 (46) | U | Y | Y | Y | Y | Y | N | Y | Y | Y |  |
| Chasco  et al. 2022 (50) | U | Y | Y | Y | Y | Y | N | Y | Y | Y |  |
| Gyllensten et al. 2023 (24) | Y | Y | Y | Y | Y | N | U | Y | Y | Y |  |
| Lunt  et al. 2024 (60) | Y | Y | Y | Y | Y | N | N | Y | Y | Y |  |
| MacEwan  et al. 2025 (61) | U | Y | Y | Y | Y | Y | N | Y | Y | Y |  |
| Miller  et al. 2024 (62) | Y | Y | Y | Y | Y | Y | U | Y | Y | Y |  |
| Nielsen  & Yarker 2023 (29) | Y | Y | Y | Y | Y | Y | Y | Y | Y | Y |  |
| Stelson  et al. 2023 (64) | Y | Y | Y | Y | Y | Y | Y | Y | Y | Y |  |
| **JBI Critical Appraisal Checklist for Cohort Studies** | | | | | | | | | | | |
| ***Authors*** | ***Q1*** | ***Q2*** | ***Q3*** | ***Q4*** | ***Q5*** | ***Q6*** | ***Q7*** | ***Q8*** | ***Q9*** | ***Q10*** | ***Q11*** |
| Altmann  et al. 2023 (45) | Y | Y | Y | N | N | U | Y | Y | Y | N/A | U |
| Ayoubkhani et al. 2024 (47) | Y | Y | Y | Y | Y | Y | Y | Y | U | N | Y |
| Brehon  et al. 2022 (18) | N/A | N/A | Y | Y | Y | N | Y | Y | Y | N/A | Y |
| Ida  et al. 2024 (51) | N/A | N/A | Y | N | N | U | Y | Y | N | N | Y |
| Frisk et al. 2023 (52) | N/A | N/A | Y | N | Y | N | Y | Y | Y | N | Y |
| Jebrini  et al. 2025 (56) | Y | Y | Y | Y | Y | N | N | Y | U | Y | Y |
| Kerksieck  et al. 2023 (57) | Y | Y | Y | Y | Y | U | Y | Y | Y | U | Y |
| Müller  et al. 2024 (37) | N/A | N/A | Y | Y | Y | U | Y | Y | Y | N | Y |
| Rutsch  et al. 2023 (17) | N/A | N/A | Y | U | Y | N | U | Y | Y | Y | Y |
| Venkatesh et al. 2024 (66) | Y | Y | Y | Y | Y | U | Y | Y | Y | N | Y |
| Westerlind et al. 2021 (68) | N/A | N/A | Y | Y | Y | N | Y | Y | Y | Y | Y |
| **JBI Critical Appraisal Checklist for Cross-sectional Studies** | | | | | | | | | | | |
| ***Authors*** | ***Q1*** | ***Q2*** | ***Q3*** | ***Q4*** | ***Q5*** | ***Q6*** | ***Q7*** | ***Q8*** |  |  |  |
| Bonner & Ghouralal, 2024 (48) | U | Y | Y | Y | Y | Y | U | Y |  |  |  |
| Braig  et al. 2024 (49) | Y | Y | Y | Y | Y | Y | Y | Y |  |  |  |
| Delgado-Alonso  et al. 2022 (12) | Y | Y | Y | Y | N | N | Y | U |  |  |  |
| Diem  et al. 2022 (15) | Y | Y | Y | Y | N | N | U | Y |  |  |  |
| Green  et al. 2024 (53) | U | Y | Y | Y | N | N | Y | Y |  |  |  |
| Harvey-Dunstan  et al. 2022 (54) | Y | Y | Y | Y | N | N | U | Y |  |  |  |
| Jaber  et al. 2025 (55) | Y | Y | Y | Y | N | N | Y | Y |  |  |  |
| Kisiel et al. 2023 (58) | U | Y | Y | Y | N | N | Y | Y |  |  |  |
| LeGoff  et al. 2023 (59) | Y | Y | Y | Y | N | N | U | Y |  |  |  |
| Saade  et al. 2024 (63) | N | Y | Y | Y | Y | Y | Y | Y |  |  |  |
| Strassburger et al. 2023 (65) | Y | Y | Y | Y | N | N | Y | U |  |  |  |
| Walker  et al. 2023 (67) | Y | Y | Y | Y | Y | Y | Y | Y |  |  |  |
| U=Unclear; Y=Yes; N=No; N/A=Not applicable | | | | | | | | | | | |

**Supplementary Table S5.** Facilitators and obstacles for work ability and RTW in individuals with post-COVID.

| 1. **Disease-related factors associated with SARS-CoV-2 infection** | |
| --- | --- |
| Facilitators | Obstacles |
| - **Higher subjective perceived physical and mental health related to post-COVID** (Müller et al., 2024▪) | - **Disease severity and course:**   - **Longer time since infection** (Ayoubkhani et al., 2024▪; Jaber et al., 2025▪)   - **ICU admission** (Braig et al., 2024▪)   - **Inpatient Treatment** (Braig et al., 2024▪; Westerlind et al., 2021▪)   - **Hospitalisation with COVID-19** (Jaber et al., 2025▪)   - **Persisting symptoms** (Ida et al., 2024▪; Kerksieck et al., 2023▪)   - **Higher PCS Score** (Kisiel et al., 2023▪)   - **>= 5 post-COVID symptoms** (Venkatesh et al., 2024▪) - **Neurological and cognitive symptoms:**   - **Neurocognitive impairment** (Altmann et al., 2023▪; Braig et al., 2024▪; Müller et al., 2024▪)   - **Cognitive symptoms** (e.g., memory problems) (Anderson et al., 2025●; Delgado-Alonso et al., 2022▪; Jebrini et al., 2025▪; Kerksieck et al., 2023▪; Lunt et al., 2024●; Nielsen & Yarker, 2023 ●; Rutsch & Deck, 2023▪; Saade et al., 2024▪; Walker et al., 2023▪)   - **Brain fog** (Chasco et al., 2022●; Green et al., 2023▪; MacEwan et al., 2025●; Miller et al., 2024●)   - **Headache/dizziness** (Braig et al., 2024▪; MacEwan et al., 2025●; Miller et al., 2024●; Venkatesh et al., 2024▪) - **Fatigue and energy-related symptoms:**   - **Fatigue** (Braig et al., 2024▪; Chasco et al., 2022●; Delgado-Alonso et al., 2022▪; Diem et al., 2022▪; Green et al., 2023▪; Gyllensten et al., 2023●; Harvey-Dunstan et al., 2022▪; Jebrini et al., 2025▪; MacEwan et al., 2025●; Miller et al., 2024●; Müller et al., 2024▪; Nielsen & Yarker, 2023 ●; Rutsch & Deck, 2023▪; Saade et al., 2024▪; Venkatesh et al., 2024▪; Walker et al., 2023▪)   - **Fluctuating symptoms** (Anderson et al., 2025●; Lunt et al., 2024●; Nielsen & Yarker, 2023●; Stelson et al., 2023●)   - **Overexertion risk** (Anderson et al., 2025●) - **Physical and somatic symptoms:**    - **Chest symptoms** (Braig et al., 2024▪)   - **Musculoskeletal pain** (Braig et al., 2024▪; Venkatesh et al., 2024▪)   - **Anosmia/Dysgeusia** (Braig et al., 2024▪)   - **Breathlessness** (Miller et al., 2024●; Venkatesh et al., 2024▪)   - **Heart palpitations** (Miller et al., 2024●)   - **Disturbance of appetite** (Jebrini et al., 2025▪) - **Mental disorder during/after SARS-CoV-2 infection** (Braig et al., 2024▪; Walker et al., 2023▪) - **Biomarkers**:   - **Elevated CRP, IL-6, leukocytes and neutrophile granulocytes, level of triglycerides** (Jebrini et al., 2025▪)   - **Lower levels in HDL-Cholesterol** (Jebrini et al., 2025▪)   - **Coagulation parameters** (Jebrini et al., 2025▪) - **Reinfection with SARS-CoV-2** (Lunt et al., 2024●) |
| 1. **Individual biopsychosocial factors** | |
| **Individual sociodemographic/ socio-economic factors** | |
| Facilitators | Obstacles |
| - **Income protection** (Lunt et al., 2024●) - **Higher income strata/socioeconomic factors** (Venkatesh et al., 2024▪; Westerlind et al., 2021▪) | - **Female sex** (Braig et al., 2024▪; Lunt et al., 2024●)   - **Perimenopausal** (Lunt et al., 2024●) - **Older age** (Braig et al., 2024▪; Jebrini et al., 2025▪; Kerksieck et al., 2023▪; Westerlind et al., 2021▪) - **Lack of financial security and financial strain:**   - **Financial concerns** (Anderson et al., 2025●; Kerksieck et al., 2023▪; Lunt et al., 2024●; MacEwan et al., 2025●; Stelson et al., 2023●)   - **Income loss** (Chasco et al., 2022●; MacEwan et al., 2025●)   - **High medical costs / lack of coverage** (Chasco et al., 2022●)   - **Statutory sick pay limits** (Anderson et al., 2025●)   - **Insurance changes 🡪 reduced coverage** (MacEwan et al., 2025●) |
| **Individual physical factors/ risk factors** | |
| Facilitators | Obstacles |
| - **Physical capacity:**   - **Better physical performance** (Ida et al., 2024▪; Müller et al., 2024▪; Strassburger et al., 2023▪)   - **Better Karnofsky Index** (Jebrini et al., 2025▪) - **General health** (Jaber et al., 2025▪) | - **Lack of physical strength** (MacEwan et al., 2025●) - **Pre-existing physical conditions** (Bonner & Ghouralal, 2024▪; Braig et al., 2024▪; Jaber et al., 2025▪; Lunt et al., 2024●) - **Current or former** **smoker** (Braig et al., 2024▪) - **Obesity/body weight and height** (Bonner & Ghouralal, 2024▪; Braig et al., 2024▪; Jebrini et al., 2025▪; Lunt et al., 2024 ●) |
| **Individual psychological/ neuropsychological factors** | |
| Facilitators | Obstacles |
| - **Microplanning cognitive demands** (Lunt et al., 2024●) - **Self-management strategies/ Pacing/ Accepting functional limitations** (Lunt et al., 2024●; Nielsen & Yarker, 2023●) - **Life-Style changes (mitigate physical risk factors)** (Lunt et al., 2024●) - **Communication strategies** (Lunt et al., 2024●) - **Coping** (Strassburger et al., 2023 ▪) - **Higher quality of life** (Green et al., 2023▪; Ida et al., 2024▪; Jebrini et al., 2025▪; Strassburger et al., 2023▪) | - **Pre-existing (neuro)psychological conditions** (Bonner & Ghouralal, 2024▪; Braig et al., 2024▪; Jaber et al., 2025▪; Kerksieck et al., 2023▪; Lunt et al., 2024●) - **Limited psychological well-being:**   - **New or worsened psychiatric diagnosis** (Kerksieck et al., 2023▪)   - **Higher levels of depressive symptoms** (Jebrini et al., 2025▪) - **Perceived stigma and social evaluation:**    - **Symptom concealment** (Anderson et al., 2025●; Chasco et al., 2022●)   - **Feeling scrutinized and under pressure** (Chasco et al., 2022●)   - **Performance pressure** (MacEwan et al., 2025●)   - **Fear of negative judgement of others** (Lunt et al., 2024●) - **General identity and self-perception:**   - **Spoiled identity: shame, guilt and self-doubt** (Anderson et al., 2025●; Chasco et al., 2022●; Miller et al., 2024●)   - **Identity conflict: neither sick nor well** (Anderson et al., 2025●)   - **Role reversal distress** (Anderson et al., 2025●) - **Emotional distress:**   - **Feelings of failure and grief** (Anderson et al., 2025●; Miller et al., 2024●)   - **Loss of routine** (MacEwan et al., 2025●) |
| **Individual social Factors** | |
| Facilitators | Obstacles |
| - **Social support (Family/Partners)** (Stelson et al., 2023●) - **Reorganisation of home responsibilities** (Stelson et al., 2023●) | - **Social withdrawal** (Anderson et al., 2025●) |
| **Individual factors related to work ability and workplace** | |
| Facilitators | Obstacles |
| - **Prioritising health over work** (Nielsen & Yarker, 2023●) - **Higher subjective perceived ability to work** (Rutsch & Deck, 2023▪) - **Higher work domain of WSAS (Work and Social Adjustment Scale)** (Walker et al., 2023▪) | - **Stigma work and society** (Anderson et al., 2025●; Chasco et al., 2022●; Lunt et al., 2024●; MacEwan et al., 2025●; Stelson et al., 2023●) - **Work identity stress, loss of capable, reliable worker identity/loss over career spirations** (Anderson et al., 2025●; Lunt et al., 2024●; MacEwan et al., 2025●; Miller et al., 2024●; Nielsen & Yarker, 2023●) - **Perceived unreliability at work** (Miller et al., 2024●) - **Emotional distress after redundancy or failed RTW** (Anderson et al., 2025●; MacEwan et al., 2025●) - **Dissatisfaction with own work ability** (Jebrini et al., 2025▪) - **Energy trade-offs: work over leisure or family** (Anderson et al., 2025●) - **Sick leave before COVID-19** (Westerlind et al., 2021 ▪) |
| 1. **Contextual workplace factors** | |
| Facilitators | Obstacles |
| - **Recognition of post-COVID as an occupational illness** (Miller et al., 2024●) - **Social and managerial support:**   - **Supportive, trusting manager/employer** (Anderson et al., 2025●; Chasco et al., 2022●; Gyllensten et al., 2023●)   - **Collegial support/encouraged culture/feeling of valuable worker** (Anderson et al., 2025●; Gyllensten et al., 2023●; Lunt et al., 2024●; Nielsen & Yarker, 2023●)   - **Employer willingness for accommodations** (Anderson et al., 2025●)   - **Job security** (Lunt et al., 2024●)   - **Instrumental support** (Nielsen & Yarker, 2023●) - **Flexibility and job/task adjustments and reorganisation:**   - **Flexible roles, hours and schedules** (Anderson et al., 2025●; Chasco et al., 2022●; Gyllensten et al., 2023●; Lunt et al., 2024●; Strassburger et al., 2023▪)   - **Adjusted working tasks** (Gyllensten et al., 2023●; Strassburger et al., 2023▪)   - **Cheat sheets/ list-making** (Chasco et al., 2022●; MacEwan et al., 2025●)   - **Frequent breaks at work** (Chasco et al., 2022●; Gyllensten et al., 2023●; MacEwan et al., 2025●; Miller et al., 2024●; Stelson et al., 2023●)   - **Remote work** (Chasco et al., 2022●; Gyllensten et al., 2023●; Lunt et al., 2024●; Miller et al., 2024●; Nielsen & Yarker, 2023●; Stelson et al., 2023●)   - **Modified duties at admission** (Brehon et al., 2022)   - **Task simplification** (Gyllensten et al., 2023●)   - **Hybrid working-models** (Miller et al., 2024●)   - **Part-time work** (Miller et al., 2024●; Nielsen & Yarker, 2023●; Stelson et al., 2023●)   - **Quiet spaces for focused work** (Miller et al., 2024●)   - **Long-term adjustments** (Gyllensten et al., 2023●)   - **Phased return-to-work program** (Miller et al., 2024●)   - **Individually tailored phase return** (Lunt et al., 2024●)   - **More hours across more days** (Lunt et al., 2024●)   - **Becoming self-employed (related to autonomy and flexibility)** (Lunt et al., 2024●)   - **Workplace adjustments** (Strassburger et al., 2023▪) - **Knowledge and awareness in the workplace: Training for employers** (Miller et al., 2024●) - **Access to occupational therapists at the workplace** (Miller et al., 2024●) | - **Visa requirements** (Anderson et al., 2025●) - **No formal post-COVID recognition → limited disability benefits** (Chasco et al., 2022●) - **Lack of social and managerial support:**   - **Lack of HR/management support** (Anderson et al., 2025●; Nielsen & Yarker, 2023●)   - **Manager distrust** (Miller et al., 2024●)   - **Isolation and not feeling part of the community** (Nielsen & Yarker, 2023●) - **Lack of knowledge and awareness in the workplace:**    - **Lacking COVID knowledge and understanding at work** (Gyllensten et al., 2023●; Lunt et al., 2024●; Miller et al., 2024●; Stelson et al., 2023●)   - **Withdrawal of accommodations** **(e.g., remote work)** (MacEwan et al., 2025●) - **Work/task-related stressors and work-organisational barriers:**   - **Rigid return policies** (Anderson et al., 2025●; Nielsen & Yarker, 2023●)   - **Unrealistic performance expectations** (Anderson et al., 2025●)   - **Multitasking** (Chasco et al., 2022●)   - **Fast-paced work environments/work stress** (Chasco et al., 2022●)   - **Post-work exhaustion** (Gyllensten et al., 2023●)   - **Night shifts** (Kerksieck et al., 2023▪)   - **Pressure to RTW** (MacEwan et al., 2025●)   - **Long daily commutes** (Miller et al., 2024●)   - **Screen work** (Nielsen & Yarker, 2023●)   - **Working with patients and pupils** (Nielsen & Yarker, 2023●)   - **Generic advices** (Nielsen & Yarker, 2023●)   - **Read dense material or produce writing** (e-mails) (Stelson et al., 2023●) |
| 1. **Healthcare system** **and service-related factors** | |
| Facilitators | Obstacles |
| - **Therapeutic support and interventions:**   - **Occupational health support** (Anderson et al., 2025●)   - **NCSE (neurocognitive screening evaluation) + mental health care** (LeGoff et al., 2023)   - **Occupational therapy** (MacEwan et al., 2025●)   - **Speech therapy** (MacEwan et al., 2025●)   - **Cognitive therapy** (MacEwan et al., 2025●)   - **Use of long-COVID clinics (multi-professional)** (Lunt et al., 2024●)   - **Supportive healthcare providers who enable sick leave and workers' compensation by diagnosing post-COVID** (Stelson et al., 2023●) - **Cooperation between internal and external serviced** (Lunt et al., 2024●; Nielsen & Yarker, 2023●) - **Self-advocacy and symptom monitoring** (Anderson et al., 2025●) - **Treatment adherence** (Lunt et al., 2024●) | - **Access to healthcare and services:**   - **More Days between symptom onset and program admission** (Brehon et al., 2022▪)   - **Higher perceived need for additional support** (Green et al., 2023▪)   - **Difficult therapy access** (Lunt et al., 2024●; MacEwan et al., 2025●) - **Administrative burden** (Anderson et al., 2025●; Chasco et al., 2022●) |
| Notes: ●=qualitative study design; ▪=quantitative study design; ICU=Intensive Care Unit; COVID=coronavirus disease; PCS=Post-COVID Syndrom; RTW=return-to-work | |

**References**

Altmann, C. H., Zvonova, E., Richter, L., & Schuller, P. O. (2023). Pulmonary recovery directly after COVID-19 and in Long-COVID. *Respiratory Physiology & Neurobiology*, *315*, 104112. https://doi.org/10.1016/j.resp.2023.104112

Anderson, E., Hunt, K., Wild, C., Nettleton, S., Ziebland, S., & MacLean, A. (2025). Episodic disability and adjustments for work: the ‘rehabilitative work’ of returning to employment with Long Covid. *Disability & Society*, *40*(5), 1239-1261. https://doi.org/10.1080/09687599.2024.2331722

Ayoubkhani, D., Zaccardi, F., Pouwels, K. B., Walker, A. S., Houston, D., Alwan, N. A.,…Nafilyan, V. (2024). Employment outcomes of people with Long Covid symptoms: community-based cohort study. *European Journal of Public Health*, *34*(3), 489-496. https://doi.org/10.1093/eurpub/ckae034

Bonner, C., & Ghouralal, S. L. (2024). Long COVID and Chronic Conditions in the US Workforce: Prevalence, Productivity Loss, and Disability. *Journal of occupational and environmental medicine*, *66*(3), e80-e86. https://doi.org/10.1097/JOM.0000000000003026

Braig, S., Peter, R. S., Nieters, A., Kräusslich, H.-G., Brockmann, S. O., Göpel, S.,…Rothenbacher, D. (2024). Post-COVID syndrome and work ability 9-12 months after a SARS-CoV-2 infection among over 9000 employees from the general population. *IJID Regions*, *10*, 67-74. https://doi.org/10.1016/j.ijregi.2023.11.015

Brehon, K., Niemeläinen, R., Hall, M., Bostick, G. P., Brown, C. A., Wieler, M., & Gross, D. P. (2022). Return-to-Work Following Occupational Rehabilitation for Long COVID: Descriptive Cohort Study. *JMIR Rehabilitation and Assistive Technologies*, *9*(3), e39883. https://doi.org/10.2196/39883

Chasco, E. E., Dukes, K., Jones, D., Comellas, A. P., Hoffman, R. M., & Garg, A. (2022). Brain Fog and Fatigue following COVID-19 Infection: An Exploratory Study of Patient Experiences of Long COVID. *International Journal of Environmental Research and Public Health*, *19*(23). https://doi.org/10.3390/ijerph192315499

Delgado-Alonso, C., Cuevas, C., Oliver-Mas, S., Díez-Cirarda, M., Delgado-Álvarez, A., Gil-Moreno, M. J.,…Matias-Guiu, J. A. (2022). Fatigue and cognitive dysfunction are associated with occupational status in Post-COVID syndrome. *International Journal of Environmental Research and Public Health*, *19*(20), 13368. https://doi.org/10.3390/ijerph192013368

Diem, L., Schwarzwald, A., Friedli, C., Hammer, H., Gomes-Fregolente, L., Warncke, J.,…Hoepner, R. (2022). Multidimensional phenotyping of the post-COVID-19 syndrome: A Swiss survey study. *CNS Neuroscience & Therapeutics*, *28*(12), 1953-1963. https://doi.org/10.1111/cns.13938

Green, C. E., Leeds, J. S., & Leeds, C. M. (2023). Occupational effects in patients with post-COVID-19 syndrome. *Occupational Medicine*, *74*(1), 86-92. https://doi.org/10.1093/occmed/kqad118

Gyllensten, K., Holm, A., & Sanden, H. (2023). Workplace factors that promote and hinder work ability and return to work among individuals with long-term effects of COVID-19: A qualitative study. *Work*, *75*(4), 1101-1112. https://doi.org/10.3233/WOR-220541

Harvey-Dunstan, T. C., Jenkins, A. R., Gupta, A., Hall, I. P., & Bolton, C. E. (2022). Patient-related outcomes in patients referred to a respiratory clinic with persisting symptoms following non-hospitalised COVID-19. *Chronic Respiratory Disease*, *19*. https://doi.org/10.1177/14799731211069391

Ida, F. S., Ferreira, H. P., Vasconcelos, A. K. M., Furtado, I. A. B., Fontenele, C. J. P. M., & Pereira, A. C. (2024). Post-COVID-19 syndrome: persistent symptoms, functional impact, quality of life, return to work, and indirect costs - a prospective case study 12 months after COVID-19 infection. *Cadernos de Saúde Publica*, *40*(2). https://doi.org/10.1590/0102-311xen026623

Jaber, F., Hoang, M. A., Feldman, D. E., Saunders, S., & Mazer, B. (2025). Factors associated with changes in employment in individuals with long COVID. *Work*, *80*(4), 1854-1860. https://doi.org/10.1177/10519815241300409

Jebrini, T., Ruzicka, M., Volk, F., Fonseca, G. J. I., Pernpruner, A., Benesch, C.,…Stubbe, H. C. (2025). Predicting work ability impairment in post COVID-19 patients: a machine learning model based on clinical parameters. *Infection*, *53*, 1189-1197. https://doi.org/10.1007/s15010-024-02459-8

Kerksieck, P., Ballouz, T., Haile, S. R., Schumacher, C., Lacy, J., Domenghino, A.,…Menges, D. (2023). Post COVID-19 condition, work ability and occupational changes in a population-based cohort. *The Lancet Regional Health*, *31*, 100671. https://doi.org/10.1016/j.lanepe.2023.100671

Kisiel, M. A., Lee, S., Malmquist, S., Rykatkin, O., Holgert, S., Janols, H.,…Zhou, X. (2023). Clustering Analysis Identified Three Long COVID Phenotypes and Their Association with General Health Status and Working Ability. *Journal of Clinical Medicine*, *12*(11). https://doi.org/10.3390/jcm12113617

LeGoff, D. B., Lazarovic, J., Kofeldt, M., & Peters, A. (2023). Neurocognitive and Symptom Validity Testing for Post-COVID-19 Condition in a Workers Compensation Context. *Journal of occupational and environmental medicine*, *65*(10), 803-812. https://doi.org/10.1097/JOM.0000000000002921

Lockwood, C., Munn, Z., & Porritt, K. (2015). Qualitative research synthesis: methodological guidance for systematic reviewers utilizing meta-aggregation. *Int J Evid Based Healthc*, *13*(3), 179-187. https://doi.org/10.1097/XEB.0000000000000062

Lunt, J., Hemming, S., Elander, J., Burton, K., & Hanney, B. (2024). Sustaining work ability amongst female professional workers with long COVID. *Occupational Medicine (Oxford, England)*, *74*(1), 104-112. https://doi.org/10.1093/occmed/kqad134

MacEwan, S. R., Rahurkar, S., Tarver, W. L., Eiterman, L. P., Melnyk, H., Olvera, R. G.,…McAlearney, A. S. (2025). The Impact of Long COVID on Employment and Well-Being: A Qualitative Study of Patient Perspectives. *Journal of General Internal Medicine*, *40*, 1070-1077. https://doi.org/10.1007/s11606-024-09062-5

Miller, A., Song, N., Sivan, M., Chowdhury, R., & Burke, M. R. (2024). Identifying the needs of people with long COVID: a qualitative study in the UK. *BMJ Open*, *14*(6), e082728. https://doi.org/10.1136/bmjopen-2023-082728

Moola, S., Munn, Z., Tufanaru, C., Aromataris, E., Sears, K., Sfetcu, R.,…Mu, P. (2020). Systematic reviews of etiology and risk. In E. Aromataris, C. Lockwood, K. Porritt, B. Pilla, & Z. Jordan (Eds.), *JBI Manual for Evidence Synthesis* (pp. 252-311). https://doi.org/10.46658/JBIMES-24-06

Müller, K., Poppele, I., Ottiger, M., Wastlhuber, A., Weber, R.-C., Stegbauer, M., & Schlesinger, T. (2024). Long-term course and factors influencing work ability and return to work in post-COVID patients 12 months after inpatient rehabilitation. *Journal of Occupational Medicine and Toxicology*, *19*(1). https://doi.org/10.1186/s12995-024-00443-4

Nielsen, K., & Yarker, J. (2023). “It’s a rollercoaster”: the recovery and return to work experiences of workers with long COVID. *Work and Stress*, *38*(2), 202-230. https://doi.org/10.1080/02678373.2023.2286654

Rutsch, M., & Deck, R. (2023). Occupational stress of Long Covid rehabilitants and return to work after pneumological rehabilitation. *Rehabilitation*, *62*(06), 369-378. https://doi.org/10.1055/a-2105-5810

Saade, A., Didier, Q., Cha, L., Garlantezec, R., Paris, C., & Tattevin, P. (2024). The prevalence, determinants, and consequences of post-COVID in healthcare workers: A cross-sectional survey. *Journal of Medical Virology*, *96*(6), e29725. https://doi.org/10.1002/jmv.29725

Stelson, E. A., Dash, D., McCorkell, L., Wilson, C., Assaf, G., Re'em, Y., & Wei, H. (2023). Return-to-work with long COVID: An Episodic Disability and Total Worker Health(R) analysis. *Social Science and Medicine*, *338*, 116336. https://doi.org/10.1016/j.socscimed.2023.116336

Strassburger, C., Hieber, D., Karthan, M., Juster, M., & Schobel, J. (2023). Return to work after Post-COVID: describing affected employees' perceptions of personal resources, organizational offerings and care pathways. *Frontiers in PublicHealth*, *11*, 1282507. https://doi.org/10.3389/fpubh.2023.1282507

Venkatesh, A. K., Yu, H., Malicki, C., Gottlieb, M., Elmore, J. G., Hill, M. J.,…Group, I. (2024). The association between prolonged SARS-CoV-2 symptoms and work outcomes. *PloS One*, *19*(7), e0300947. https://doi.org/10.1371/journal.pone.0300947

Walker, S., Goodfellow, H., Pookarnjanamorakot, P., Murray, E., Bindman, J., Blandford, A.,…Collaboration, L. (2023). Impact of fatigue as the primary determinant of functional limitations among patients with post-COVID-19 syndrome: a cross-sectional observational study. *BMJ Open*, *13*(6), e069217. https://doi.org/10.1136/bmjopen-2022-069217

Westerlind, E., Palstam, A., Sunnerhagen, K. S., & Persson, H. C. (2021). Patterns and predictors of sick leave after Covid-19 and long Covid in a national Swedish cohort. *BMC Public Health*, *21*(1), 1023. https://doi.org/10.1186/s12889-021-11013-2
